# Iron overload reprogramming lipid metabolism through the IRP1–SCAP axis in fibroblast-like synoviocytes aggravates bone destruction in rheumatoid arthritis

**DOI:** 10.1038/s12276-026-01710-6

**Published:** 2026-05-01

**Authors:** Yan Liu, Linkai Fang, Manli Wang, Shuyuan Zhong, Yiding Xiong, Yuluan Hou, Jianhua Ren, Yuhang Li, Weihang Zhu, Xia Meng, Chenyang Lu, Yunfeng Pan

**Affiliations:** 1 Department of Rheumatology and Immunology, The Third Affiliated Hospital of Sun Yat-sen University, Guangzhou, China; 2 Division of Rheumatology and Immunology, Department of Immunology, School of Cell and Gene Therapy, Songjiang Research Institute, Shanghai Songjiang District Central Hospital, Shanghai Jiao Tong University School of Medicine, Shanghai, China; 3 Department of Stomatology, The Third Affiliated Hospital of Sun Yat-sen University, Guangzhou, China; 4 Department of Joint and Trauma Surgery, The Third Affiliated Hospital of Sun Yat-sen University, Guangzhou, China

**Keywords:** Rheumatoid arthritis, Cell invasion

## Abstract

Rheumatoid arthritis (RA) is a leading cause of disability globally. Although iron accumulation in arthritic lesions has been observed in patients with RA, its specific contribution to disability outcomes remains unclear. Here we demonstrate a comprehensive multiomics approach to elucidate the impact and underlying mechanisms of iron overload in RA. First, clinical radiology in an RA cohort reveals a positive correlation between elevated ferrous iron levels in synovial fluid and joint damage extent. Iron chelator DFO administration significantly alleviates bone destruction in the K/BxN serum-transfer induced arthritis mice model. In terms of cellular function, we identify the aggressive migration and invasion of fibroblast-like synoviocytes (FLSs) induced by excess iron utilizing a humanized synovitis model. Mechanistically, the multiomics integration of transcriptomics and metabolomics indicates the enriched lipid synthesis pathway in the FLS response to iron exposure. The lipid transcription factor SREBP1 is particularly highly expressed in RA-FLSs, and its genetic ablation or pharmacological inhibition markedly mitigates the pathogenic effects of iron overload both in vitro and in vivo. At the molecular level, iron regulatory protein IRP1 enhances the translation of SREBP1 adapter protein SCAP by disengaging from its mRNA 5′ untranslated region upon iron stimulation. This process facilitates SREBP1 cleavage and activation, driving the upregulation of genes involved in fatty acid and cholesterol biosynthesis. Our findings elucidate the IRP1–SCAP axis as a critical modulator of lipid metabolic reprogramming in aggressive FLSs, underscoring its potential as a therapeutic target for RA by modulating the ‘iron–lipid’ crosstalk.

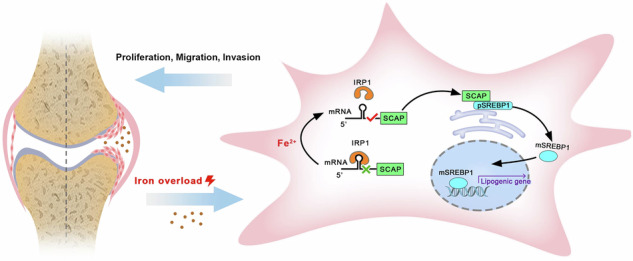

## Iron overload drives joint damage in rheumatoid arthritis

Rheumatoid arthritis (RA) is characterized by synovial hyperplasia and bone erosion, driven by fibroblast-like synoviocytes (FLSs) that exhibit aggressive, neoplastic-like behavior. This study investigates the role of iron overload in RA pathogenesis, focusing on its impact on FLSs. Using K/BxN serum-transfer induced arthritis and humanized synovitis mouse models, the authors demonstrate that elevated ferrous iron enhances FLSs proliferation and invasion via SREBP1-mediated lipogenesis. They identify that iron regulatory protein IRP1 binds to SCAP mRNA, facilitating its translation and subsequent SREBP1 activation under iron-replete conditions. This activation upregulates genes involved in lipid biosynthesis, fueling FLS aggression. The study reveals that iron chelation with deferoxamine mitigates arthritis progression by modulating local pathological processes. These findings highlight the IRP1–SCAP interaction as a potential therapeutic target, suggesting that disrupting this ‘iron–lipid’ crosstalk could offer new strategies for RA treatment.

This summary was initially drafted using artificial intelligence, then revised and fact-checked by the author.

## Introduction

Synovial hyperplasia and pannus formation are hallmark pathological features of rheumatoid arthritis (RA)^1^. Fibroblast-like synoviocytes (FLSs), the predominant mesenchymal cells in synovial tissue, exhibit neoplastic-like characteristics, including resistance to apoptosis, uncontrolled proliferation, chronic inflammation and enhanced migration and invasion^2^. These aggressive behaviors drive synovial hyperplasia and subsequent bone erosion, creating a high energy demand supported by vigorous metabolism, such as glycolysis, mitochondrial oxidative phosphorylation, lipogenesis and amino acid synthesis^3,4^. Therefore, elucidating and targeting the metabolic dysregulation in FLSs may open a promising therapeutic avenue to disrupt the inflammatory and destructive cascade in RA.

Synovial cells are driven into the pathogenic activated states by multiple stimuli in the adverse microenvironment of RA. Among these, aberrant iron levels have attracted considerable attention. Iron, as an essential trace element for cellular functions, plays a critical role in the development of the nervous, cardiovascular, hematological and immune systems^5^. However, disruptions in iron homeostasis are implicated in a variety of pathological conditions, including ischemia–reperfusion injuries^6^, degenerative diseases^7^ and metabolic disorders^8^, primarily owing to the peroxide damage and proinflammatory effects induced by excess iron. Our previous research revealed distinct ferroptosis outcomes in M1-like and M2-like macrophage subsets following iron overload in joints, exacerbating local immune disorders in RA^9^. However, the specific effects of excess articular iron on FLSs, particularly its role in synovial proliferation and bone destruction in RA, remain to be determined. Understanding whether iron-immersed FLS contribute to these pathological processes and elucidating how iron influences the metabolic characteristics of RA-FLSs require further investigation.

In the present study, we explore the impact of iron overload on FLSs in RA pathogenesis, utilizing the K/BxN serum-transfer induced arthritis (STIA) and humanized synovitis mouse models. Our findings reveal that the elevated ferrous iron promotes RA-FLSs hyperplasia and invasion by activating SREBP1-mediated de novo lipogenesis. Mechanistically, we identify for the first time that the iron regulatory protein IRP1 binds to the 5′ untranslated region (5′ UTR) of sterol regulatory element-binding protein (SREBP) cleavage-activating protein (SCAP) mRNA, an adapter protein crucial for SREBP1 cleavage, translocation and activation. Under iron-replete conditions, IRP1 dissociates from SCAP mRNA, enhancing its translation and subsequently activating SREBP1. This process transcriptionally upregulates downstream genes involved in fatty acid and cholesterol biosynthesis to fuel the aggressive capacity of RA-FLSs. These discoveries highlight IRP1–SCAP interaction as a pivotal signal nexus linking extracellular iron overload to intracellular metabolic reprogramming in FLSs, and this ‘iron–lipid’ crosstalk represents a promising therapeutic strategy for RA.

## Methods

### Patients

Human specimen research was approved by the Medical Ethics Committee of the Third Affiliated Hospital of Sun Yat-sen University (RG2023-016). All participants had signed the informed consent according to the principles illustrated in the Declaration of Helsinki. All patients including those with RA, osteoarthritis (OA) and gout arthritis (GA) were diagnosed according to the criteria of the American College of Rheumatology. The synovial fluid (SF) samples were collected during joint aspiration, and the biopsies were obtained from joint arthroscopy or synovectomy of the knee. Normal synovial tissues and cartilages samples were collected from patients who underwent surgery for traumatic injuries, providing a relative healthy control (HC) group in the study. For the iron quantification assays in joint fluid, the following patients were included: RA *n* = 15, GA *n* = 8 and OA *n* = 8. For immunohistochemistry study using human synovial biopsy tissue, the following patients were included: RA *n* = 10 and HC *n* = 10. For magnetic resonance imaging (MRI) analysis, the patients with RA who had experienced knee MRI (*n* = 11) were recruited. For the isolation of FLSs from synovial tissues, the patients with active RA (*n* = 6) were included. Detailed demographic and clinical characteristics are presented in Supplementary Tables 1–4.

### Mouse model

C57BL/6 mice and the severe combined immunodeficiency (SCID) mice were purchased from Guangdong Medical Laboratory Animal Center (Guangzhou, China). K/BxN mice sera for the establishment of STIA mode were kindly gifted by the Song Guo Zheng group from the Shanghai Jiao Tong University. All mice were housed at a barrier- and specific pathogen-free facility at the Sun Yat-sen University. Food and water were freely accessible.

The STIA model was induced as described previously^10^. In brief, 100 μl arthritogenic K/BxN mice sera were administered intraperitoneally to 8-week-old female C57BL/6 mice on day 0 and day 2. Arthritis severity was evaluated daily using a semiquantitative scoring system ranging from 0 to 4, where 0 means no evidence of erythema and swelling, 1 means mildly detectable swelling in a single digit, 2 means mild swelling extending to more than one digit, 3 means moderate swelling of the ankle and digits and 4 means severe swelling encompassing the ankle, paw and digits. All assessors were blinded to the group conditions. At day 9, mice were killed, and samples were collected for following experiments. Each animal was considered as an individual experimental unit.

The humanized synovitis mouse model was utilized to assess the migration and invasion of FLSs in vivo. The procedure was conducted in 8-week-old female SCID mice. Initially, a piece of fresh human cartilage (obtained from trauma patients undergoing joint replacement surgery with informed consent) was cut to dimensions of 0.5 × 0.4 × 0.2 cm³. Separately, a piece of sterilized, synthetic gelatin sponge (Oneshine, VG-MJHM-A) was cut to 0.7 × 0.6 × 0.6 cm³. The cartilage piece was then inserted into a slit made in the gelatin sponge, creating a stable, sandwich-like ‘cartilage–gelatin composite’ for subcutaneous implantation into the left back of SCID mice. After 14 days, another sponge including normal human cartilage was be inserted subcutaneously into the right side of the SCID mouse. After being inserted, RA-FLSs (5 × 10^5^ cells) preconditioned with ferrous sulfate (FeSO_4_) or infected with SREBP1-short hairpin RNA (shRNA) lentivirus were injected into the sponge. At day 60 post implantation, the implants were retrieved and subjected to hematoxylin and eosin (H&E) staining for histological evaluation. Two researchers were invited to perform a double-blind assessment of FLS invasion and cartilage degradation.

### FLSs isolation and culture

Synovium biopsies tissues from patients with RA were dissected free of fat and blood vessels, then finely minced into small pieces and transferred to culture flasks. The culture medium consisted of high-glucose Dulbecco’s modified Eagle medium (DMEM; Gibco), supplemented with 10% fetal bovine serum (FBS; Gibco), 100 U/ml penicillin (Gibco) and 100 μg/ml streptomycin (Gibco). The cultures were maintained in a thermostatically controlled incubator with a 5% CO_2_ atmosphere at 37 °C. Within 3–5 days, single cells crawled out from the tissue edges and progressively covered the flask. FLSs from passages 3–6 were used for subsequent experimental procedures.

### Iron quantification assays

SF samples were centrifuged at 1000*g* for 10 min, and the supernatant was isolated for iron concentration quantification using an iron assay kit (Abcam, ab83366) following the manufacturer’s instructions. The output was assessed on a colorimetric microplate reader (optical density of 593 nm).

### MRI assessment

The MRI scanning of RA knee joint in the supine position was performed with a 3.0T MRI scanner (GE Healthcare, MR750). The MRI protocol encompassed the following sequences: (1) sagittal T1-weighted imaging: section thickness 4 mm, section gap 0.4 mm, number of sections 21, field of view 180 × 180 mm^2^, voxel sizes 0.6 × 0.6 × 4 mm^3^, repetition time 771 ms, echo time 11.2 ms and acquisition time 1 min 16 s; (2) sagittal T1-weighted imaging with fat suppression and contrast enhancement (T1-FS + C): section thickness 4 mm, section gap 0.4 mm, number of sections 21, field of view 180 × 180 mm^2^, voxel sizes 0.6 × 0.6 × 4 mm^3^, repetition time 607 ms, echo time 13 ms and acquisition time 1 min 35 s; and (3) sagittal proton density-weighted imaging with fat suppression (PD-FS): section thickness 4 mm, section gap 0.4 mm, number of sections 21, filed of view 180 × 180 mm^2^, voxel sizes 0.6 × 0.6 × 4 mm^3^, repetition time 2548 ms, echo time 40 ms, acquisition time 1 min 47 s. The semiquantitative scoring of synovitis, bone erosion and bone marrow edema was conducted in accordance with the RA MRI Scoring method^11,12^. Synovitis and synovial volume were assessed on T1-FS + C images with the scores of 0–3 (0, normal; 1, mild; 2, moderate; and 3, severe). Bone destruction was evaluated on T1-weighted images and scored on an erosion scale ranging from 0 to 10, with increments of 10% erosion presence. Bone marrow edema was assessed on PD-FS images, with a scoring system from 0 (normal) to 3 (severe). All MRI parameter evaluations were performed utilizing the ITK-SNAP 3.5 workstation.

### Histological evaluation

The hind limbs of mice with induced arthritis were collected and fixed in 4% paraformaldehyde. Decalcification was achieved using ethylenediaminetetraacetic acid (EDTA). Subsequently, the specimens were embedded in paraffin and cut into 4-μm-thick sections for following staining. H&E (Service Bio) was used to evaluate synovitis severity using a semiquantitative grading scale: 0, no signs of inflammation; 1, mild inflammation with hyperplasia of the synovial lining; 2, moderate infiltration with noticeable synovial hyperplasia; 3, marked infiltration with significant synovial hyperplasia; and 4, severe inflammatory cell infiltration and pronounced synovial hyperplasia. Toluidine blue (TB) staining was utilized to assess cartilage destruction, whereas tartrate-resistant acid phosphatase (Trap) staining was conducted to quantify osteoclast distribution. All histological slides were evaluated by investigators blinded to the experimental conditions.

### Quantitative rtPCR

Total RNA was isolated from samples using the RNA-Quick Purification Kit (ESScience, ES-RN001) according to the manufacturer’s protocol. Complementary DNA (cDNA) was synthesized utilizing the PrimeScript RT reagent Kit (ESScience, ES-RT001). Quantitative real-time PCR (rtPCR) was conducted to determine mRNA levels with the Super SYBR Green quantitative PCR (qPCR) Master Mix Kit (ESScience, ES-QP002) on a Quant Studio 5 (ABI) qPCR machine. All primers were synthesized by Tsingke Biotechnology. The primer sequences are detailed in Supplementary Table 5.

### Western blot analysis

Total protein was extracted from cells or tissues with RIPA lysis buffer (Sigma, R0278). Proteins were conducted using sodium dodecyl sulfate–polyacrylamide gel electrophoresis, followed by detection with the enhanced chemiluminescent system (Epizyme, SQ101). The primary antibodies used were as follows: rabbit anti-SREBP1 (Proteintech, 14088-1-AP), rabbit anti-SREBP2 (Proteintech, 28212-1-AP), rabbit anti-CYP51A1 (Proteintech, 13431-1-AP), rabbit anti-FASN (Proteintech, 10624-2-AP), rabbit anti-HMGCR (Zen BioScience, R24588), rabbit anti-ACLY (Zen BioScience, R23558), rabbit anti-INSIG1 (Proteintech, 55282-1-A), rabbit anti-INSIG2 (Proteintech, 24766-1-AP), rabbit anti-IRP1 (Proteintech, 12406-1-AP), rabbit anti-IRP2 (Proteintech, 23829-1-AP), rabbit anti-SCAP (Bethyl, A303-554A) and mouse anti-ACTB/β-actin (Proteintech, 20536-1-AP). Immobilized images were quantified using ImageJ software (National Institutes of Health).

### IF staining

FLSs seeded on coverslips were fixed with 4% paraformaldehyde and permeabilized using 0.25% Triton X-100. After blocking with 5% bovine serum albumin, the sections were incubated with the primary antibodies at 4 °C overnight, then stained with the corresponding secondary antibodies for 1 h at room temperature and mounted with DAPI Fluoromount (Abcam, ab188804). The primary antibodies used were rabbit anti-SREBP1 (Proteintech, 14088-1-AP,1:500), rabbit anti-SREBP2 (Proteintech, 28212-1-AP, 1:500) and rabbit anti-IRP1 (Proteintech, 12406-1-AP, 1:500). Alexa Fluor 488 conjugated goat anti-rabbit IgG (Invitrogen, A11008, 1:2000) and Alexa Fluor 555 conjugated goat anti-rabbit IgG (Invitrogen, A31572, 1:2000) were used as the secondary antibodies. Image analysis was performed using the NIH ImageJ software.

### Cell viability, migration and invasion assays

Cell viability was determined by the cell counting kit 8 (CCK-8) (Dojindo, CK04) according to the manufacturer’s instructions. To evaluate FLS migration, a Transwell system with 8-μm-pore-diameter membrane inserts (BD Falcon) was used. In brief, 1 × 10^4^ FLSs suspended in serum-free DMEM were seeded into the upper chamber, whereas DMEM supplemented with 10% FBS was placed in the lower compartment to serve as a chemoattractant. After a 24-h incubation, cells that had penetrated the filter were fixed with 4% paraformaldehyde and stained with 0.1% crystal violet. For the cell invasion assay, Matrigel matrix (Corning, 356234) diluted with serum-free DMEM was precoated onto the Transwell inserts, and FLSs that transferred through the membrane were stained using crystal violet, followed by photographing and quantification.

### Flow cytometry analysis

Cell death in FLSs was assessed using Fixable Viability Dye eFluor 780 (eBioscience, 65-0865-14) according to the manufacturer’s guidelines. Cell cycle progression was analyzed with Cell Cycle Analysis Kit (ESScience, ES-CC001) according to the manufacturer’s protocol. Data acquisition was performed using a flow cytometer, and subsequent analysis of the results was conducted using FlowJo software.

### Protein array

Biological makers in the joint of STIA mice was assessed by Proteome Profiler Mouse Array Kit (R&D system, ARY015). The protein of paw tissues was extracted with RIPA lysis buffer (Sigma, R0278) and then analyzed with the protein array. The expression levels of each target protein were quantified using ImageJ software (National Institutes of Health) and normalized to the corresponding reference spots on the array.

### Inflammatory cytokines measurement

Serum from STIA mice was collected via centrifugation at 15,000*g* for 15 min. The levels of proinflammatory cytokines TNF-α, IL-1β and IL-6 in serum samples were quantified using the enzyme-linked immunosorbent assays (ELISA) kits (DAKEWE, 1217202, 1210122 and 1210602) following the manufacturer’s instructions.

### Lipid substance detection

The concentrations of lipids in FLSs and SF were measured using commercial free fatty acid (FFA), triglyceride (TG) and total cholesterol quantification kits following the manufacturer’s protocols (Njjcbio, A042-2-1, A110-1-1, A111-1-1). Filipin III, a fluorescence dye (Cayman, 70440), was used to detect cholesterol in FLSs after fixation with 4% paraformaldehyde. Cellular neutral lipid content was visualized using Bodipy 493/503 staining (GlpBio, GC42959).

### Bulk RNA-seq and scRNA-seq analyses

FLSs from three independent patients with RA were subjected to whole transcriptome sequencing. In brief, total RNA were isolated from 2 × 10^5^ FLSs treated with 500 μM FeSO_4_ or phosphate-buffered saline (PBS) for 48 h using Trizol (Invitrogen) and then were subjected to RNA sequencing (RNA-seq) conducted by Novogene using an Illumina sequencer. Raw reads were aligned to a human reference genome (GRCh38; hg38) using Hisat2 software. Differential expression analyses were performed using the DESeq2 package in R (version 4.3.2). Genes with a *P* value <0.05 and |log_2_(fold change)| >0.25 were considered differentially expressed genes (DEGs). A volcano plot showing DEGs was graphed with the ggplot2 package. Genome ontology and signaling pathways for upregulated DEGs were analyzed with the clusterProfiler package^13^ and visualized using the Dotplot function. For specific genes, heat maps were graphed using the webtool clustervis (https://biit.cs.ut.ee/clustvis/). Transcription factors for Fe^2+^-upregulated genes were predicted using the webtool Enrichr (https://maayanlab.cloud/Enrichr/) by querying the TRRUST database^14^. Single-cell RNA-seq (scRNA-seq) datasets on RA synovial cells^15^ were subjected to further analysis utilizing the R package Seurat (version 4.1.1) following the tutorial^16^. The expression of SREBP1 and SREBP2 across synovial cells was visualized using the Dotplot function, and gene set enrichment analyses were performed with the clusterProfiler package on the basis of the MSigDB databases (https://www.gsea-msigdb.org/gsea/msigdb).

### Liquid chromatography–mass spectrometry analysis

Metabolites from RA-FLSs treated with either FeSO_4_ or PBS were extracted by vigorous shaking with precooled 80% methanol and 0.1% formic acid. An equivalent volume of the metabolite extract from each sample was pooled to serve as a quality control sample. The relative quantification of the metabolites was conducted using a Vanquish UHPLC system (Thermo Fisher Scientific) coupled to an Orbitrap Q Exactive mass spectrometer (Thermo Fisher Scientific). Nontargeted metabolite analysis was performed in both positive and negative ionization modes, followed by metabolite identification and quantification by comparison with reference databases on the basis of retention time and mass-to-charge ratio (*m*/*z*). The final metabolomic dataset was obtained after background ion subtraction using a blank sample matrix and normalization of the data.

### Gene knockdown and overexpression

Transfection of small interfering RNAs (siRNAs) into FLSs: 1.5 × 10^5^ FLSs were seeded in six-well plates and cultured to 70% confluence. After a 24-h incubation period, cells were transfected with siRNAs using the jetPRIME reagent (Polyplus-transfection, 101000046) following the manufacturer’s protocol. The sequences of three distinct siRNAs targeting iron regulatory protein 1 (IRP1) and iron regulatory protein 2 (IRP2) mRNA are presented in Supplementary Table 6.

SREBP1-shRNA lentivirus was constructed and packaged with the lentiviral transfer vector LV-3 (pGLVH1/GFP + Puro) by GenePharma. Three shRNA was designed and two efficiently knocked down SREBP1 expression, whose sequences are detailed in Supplementary Table 6.

### RIP assay

RNA immunoprecipitation (RIP) assays were conducted with the Magna RIP RNA-Binding Protein Immunoprecipitation Kit (Millipore, 17-700). In brief, RA-FLSs at a concentration of 1 × 10^7^ cells were lysed in a buffer supplemented with protease and RNase inhibitors to prevent degradation. The cell lysates were incubated with protein A/G magnetic beads conjugated to the antibody of IRP1 at 4 °C overnight, allowing for the capture of RNA–protein complexes. After the immunoprecipitation, the magnetic beads were washed to remove unbound proteins and RNA, and the associated RNA was extracted, then reverse-transcribed and quantified by reverse-transcription qPCR. The primer sequences used for the reverse-transcription qPCR are detailed in Supplementary Table 5.

### RNA pulldown assay

RNA pulldown assay was conducted using the commercial RNA Pulldown Kit (BersinBio, Bes5102) in accordance with the manufacturer’s protocol. Biotinylated RNA probes were incubated with magnetic beads for efficient binding. This complex was then incubated with the protein lysate from cells at 25°C with gentle rotation for 2 h. After five washes, the bound proteins were eluted and analyzed by western blot. The biotinylated probes used in this study were specific to the 5′ UTR of the SCAP mRNA, with the sequence 5′-GGG CAC CCG GCG GCC AGG AGA GAG AGG GAG GGC GCC ACG CAC CGG ACT GCG GGC CGA GAG CGC GCA CGC CGC GCT CCG CCC CTG CTG CCG CCC CCG TCG CCG CCG CCG CCG CCG CCG CAG CTT GGG AGG TGC TGC CAC CAC AGG TAC CTG CAC ATG TTG TTC TTT GTC AGT GCT GTC AAG TGT GTG CCA GGG TGA TCC ATG GTC ACT TTC CGG GAT GGC AGC AAG GTG ACT TCG GCT GAG G-3′, and negative control (NC) LacZ probe, with the sequence 5′-TGG CCG TCG TTT TAC AAC GTC GTG ACT GGG AAA ACC CTG GCG TTA CCC AAC TTA ATC GCC TTG CAG CAC ATC CCC CTT TCG CCA GCT GGC GTA ATA GCG AAG AGG CCC GCA CCG ATC GCC CTT CCC AAC AGT TGC GCA GCC TGA ATG GCG AAT GGC GCC TGA TGC GGT ATT TTC TCC TTA CGC ATC TGT GCG GTA TTT CAC ACC GCA TAT GGT GCA CTC TCA GTA CAA TCT GCT CTG ATG CCG CAT AG-3′. These probes were synthesized by BersinBio and used to enrich for proteins that specifically interact with the SCAP 5′ UTR.

### FISH–IF

RNA fluorescence in situ hybridization (FISH) was performed on RA-FLSs cultured on glass slides using the RNA FISH kit from BersinBio according to the manufacturer’s guidelines. The cells were fixed with 4% paraformaldehyde for 30 min at room temperature, permeabilized with 0.5% Triton X-100 and then dehydrated through a series of graded ethanol washes. Following a 30-min prehybridization at 4 °C in prehybridization buffer, the slides were hybridized with a buffer containing 6-carboxyfluorescein (FAM)-labeled probes specific to the SCAP 5′ UTR (sequence 5′-GAC GTC TGG TTC TCA GGT AGC TTA GGG GCA TCA GGT GGG AAG ATG GAG AAG GCA GGG TCC GGG TGG CTG GGG GGC AGC ATG CCA CTA GGC ACG GGC ATG GGA GCC AGG GCT CCC TCA CCC AAT GGG CTC TGT TCC GTC ACC TGG GCA GCG AGG TAG TTG CGC AGC CCT GCT GGG TCT GTG TAT ACC AGG ATG CCA ATC CAG ACA ACG GTG C-3′) provided by BersinBio for 5 min at 73 °C, followed by an overnight incubation at 37 °C. Post-hybridization washes were conducted sequentially in 4× saline sodium citrate (SSC), 2× SSC and 1× SSC at 42 °C. Immunofluorescence (IF) was performed using a rabbit anti-IRP1 primary antibody (Proteintech, 12406-1-AP) and an Alexa Fluor 555 goat anti-rabbit IgG secondary antibody (Invitrogen, A31572, diluted 1:2000). After counterstaining with DAPI to visualize nuclei, the cells were imaged using a confocal laser scanning microscope.

### Molecular docking prediction

Molecular docking predictions were performed using AlphaFold3. Initially, protein and RNA sequences were encoded into structured feature via the Input Feature Embedder, capturing the essential characteristics of amino acids and nucleotides. Pairwise representations were then constructed to define the interactions between proteins and nucleic acids, followed by further refinement through the conditioning network. RNA-binding motifs within the protein and the complementary regions of mRNA were identified and analyzed to predict possible interaction sites. Molecular docking simulations were conducted to assess the RNA-binding sites, hydrogen bonds and steric complementarity, with the results visualized using PyMOL.

### EMSA

To test the binding activity of IRP1 to SCAP 5′ UTR, an electrophoretic mobility shift assay (EMSA) was performed using a RNA EMSA kit (Bes5107, Bersinbio) according to the manufacturer’s instructions. For EMSA, the probes (sequence 5′-CUUUGUCAGUGCUGUCAAGUGUGUGCC-3′) were heated at 95 °C for 3 min, then slowly cooled to room temperature, annealed to form a stem-loop structure and then incubated with 10 μg of HIS-IRP1 for 30 min at 25 °C. Cold competitor probes (the specific competitor probe sequence 5′-CUUUGUCAGUGCUGUCAAGUGUGUGCC-3′; the mutant competitor probe sequence 5′-CUUUGUGUCACGUGUCAAGUGUGUGCC-3′) at 50× were used to confirm the binding specificity. Samples were separated using 6% native polyacrylamide gel, then transferred to nylon membrane and crosslinking by UV. Transferred probes were detected with horseradish peroxidase (HRP)-conjugated streptavidin.

### Ethics

Human specimen research was approved by the Medical Ethics Committee of the Third Affiliated Hospital of Sun Yat-sen University (RG2023-016). All participants had signed the informed consent according to the principles illustrated in the Declaration of Helsinki. All animal experiments were conducted in accordance with the relevant guidelines and regulations approved by the Institutional Animal Care and Use Committees of Guangdong Laboratory Animals Monitoring Institute (IACUC2019022).

### Statistics

Data are presented as the mean ± standard deviation (s.d.) and were analyzed using GraphPad Prism software (version 9.0). For statistical comparisons between two groups, the Student’s *t*-test was used. When comparing three or more groups, one- or two-way analysis of variance (ANOVA) was utilized without any matching or pairing. To account for multiple comparisons, Turkey’s test was applied to adjust the results. Correlations between pairs of datasets were determined using Pearson’s correlation coefficients. Error bars in the figures indicate standard deviation. A *P* value <0.05 was considered statistically significant for all analyses.

## Results

### Arthritic iron overload promotes synovium hyperplasia and bone destruction in RA

**Fig. 1:**
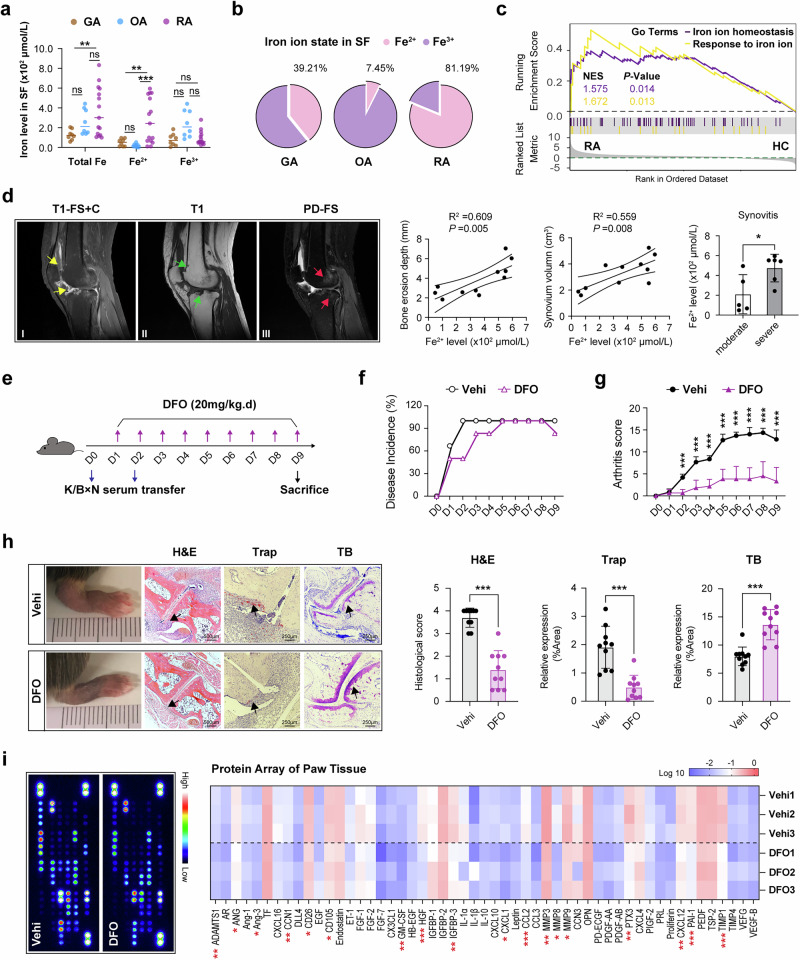
Focal iron accumulation aggregates joint destruction in RA. **a** The iron evaluation in SF of patients with RA (*N* = 15), GA (*N* = 8) and OA (*N* = 8). *P < 0.05, **P < 0.01, ns, not significant, by one-way ANOVA (mean ± s.d.). **b** The average frequencies of different iron ions status in RA SF (*N* = 15), with GA (*N* = 8) and OA (*N* = 8) used as control groups. **c** A gene set enrichment analysis enrichment in GSE89408 datasets showing that different genes between RA and HC synovial tissues were related to iron ion homeostasis (NES = 1.575, *P* = 0.014) and response to iron ion pathway (NES = 1.672, *P* = 0.013). NES, Normalized Enrichment Score. **d** Representative MRI images of the knee in patients with typical RA. I, synovium emphasized by yellow arrowhead on T1-FS + C; II, bone destruction indicated by green arrowhead on T1; III, bone marrow edema pointed by red arrowhead on PD-FS. The association of the altered ferrous iron level and the radiological indications (bone erosion depth and synovial volume) was evaluated with Spearman correlation analysis (*N* = 11), *P < 0.05. The comparison of ferrous iron level in RA SF with distinct synovitis by Student’s *t*-test. *P < 0.05; moderate synovitis, *N* = 5 patients with RA; severe synovitis, *N* = 6 patients with RA. **e** The timeline design of STIA models with DFO administration. **f** Disease incidence at various time points. **g** Arthritis severity scores were determined daily after modeling and statistically analyzed for 9 days. *N* = 10 in each group, ***P < 0.001 versus vehicle (Vehi) group by Student’s *t*-test (mean ± s.d.). **h** STIA mice with DFO injection daily were killed at day 9. Histological sections of whole paws were collected for H&E, Trap and TB analysis (the pathologic change emphasized by black arrowhead). *N* = 10 in each group. ***P < 0.001 versus Vehi group by Student’s *t*-test (mean ± s.d.). **i** The expression of biological factors in STIA paw tissues was measured using a protein array. The image density of each target was normalized to the reference of each sample and concerted to the fold change of Vehi group. Left: representative dot blots. Right: a quantitative heat map. *N* = 3 in each group. *P < 0.05, **P < 0.01, ***P < 0.001; by Student’s *t*-test (mean ± s.d.). T1, T1-weighted images.

Emerging evidence has indicated excess iron deposition in the joints of patients with RA. To elucidate the specific ionic state and its pathological role in RA progression, we quantified iron levels in the SF of RA using a colorimetric assay. SF samples from patients with GA and OA were utilized as disease controls, as arthrocentesis is not typically performed on healthy individuals. The results revealed higher levels of free iron in RA SF compared with both GA and OA groups (Fig. 1a), with a significantly notable predominance of ferrous iron (Fig. 1b). Synovial RNA-seq datasets (GSE89408) showed that genes associated with iron ion homeostasis and response to iron ion pathway, including B2M, HIF1A, SLC39A8 and HEPHL1 were markedly upregulated in synoviums from patients with RA, compared with those from HCs (Fig. 1c and Supplementary Fig. 1). Consistently, we observed aberrant expression of iron regulatory proteins in RA synoviums, characterized by a downregulation of the iron absorption molecule transferrin receptor (TFRC), upregulation of the iron excretion mediator ferroportin (FPN) and increased levels of iron chelation proteins ferritin light chain (FTL) and ferritin heavy chain 1 (FTH1), compared with the control synoviums from trauma patients (Supplementary Fig. 2). These findings indicate the direct impact of articular iron overload on RA synovial tissues.

Considering the pivotal role of synovium in mediating joint destruction, we assessed the relationship between local iron content and radiological features of patients with RA using Spearman’s correlation analysis. A positive linear correlation was found between elevated ferrous iron and synovial volume on T1-FS + C, as well as bone erosion depth on T1-weighted sequence with MRI assessment (Fig. 1d). In addition, patients with severe synovitis exhibited higher iron accumulation in SF compared with those with moderate synovitis (Fig. 1d). However, the bone marrow edema on PD-FS sequence does not differ with changes in iron concentration (data not shown). These results suggest that local iron may contribute to structural destruction of the joint in patients with RA.

To investigate the pathogenic role of iron overload in arthritis, we used the iron chelator deferoxamine (DFO) in a K/BxN STIA model. Arthritis was induced in C57BL/6 mice by intraperitoneal injection of K/BxN serum (100 μl) on two alternate days. First, DFO (20 mg/kg, intraperitoneal) was administered daily starting on the day after the first serum injection and continuing for 9 days (Fig. 1e). This preventive DFO treatment obviously reduced both disease incidence and clinical arthritis scores compared with the vehicle control (Fig. 1f,g). The histological analysis of ankle joints confirmed a marked amelioration of synovitis, synovial hyperplasia and pannus formation, along with a pronounced reduction in cartilage damage and bone erosion (Fig. 1h). Notably, however, this protective effect occurred without a significant reduction in the levels of circulating inflammatory cytokines (IL-1β, IL-6 and TNF-α) in the sera (Supplementary Fig. 3). Given this discrepancy between local joint protection and unchanged systemic cytokines, we next analyzed the local expression of pathogenic mediators within paw tissues using a protein array. As expected, we found that preventive DFO treatment substantially downregulated the levels of key chemokines (CCL2, CXCL1 and CXCL12), angiogenic factors (ANG and Ang-3) and matrix-degrading enzymes (MMP3, MMP8, MMP9 and ADAMTS1) (Fig. 1i).

We then assessed whether DFO could also confer therapeutic benefits after disease onset. A separate cohort of mice received their first DFO injection on day 3 after the second serum administration, at which point there was no baseline difference in arthritis severity between the treatment and control groups. Consistent with the preventive findings, this therapeutic DFO administration strikingly attenuated subsequent arthritis progression (Supplementary Fig. 4). The immunohistochemical analysis of joints from these mice demonstrated a significant decrease in the infiltration of macrophages and neutrophils, alongside reduced local expression of the inflammatory cytokines IL-1β and TNF-α (Supplementary Fig. 5), confirming its therapeutic efficacy at the cellular level.

Collectively, these findings suggest that SF iron overload is critically involved in the pathological inflammation and bone destruction in RA. Iron chelation with DFO, whether administered as a prevention or a therapeutic intervention, mitigates arthritis progression. This effect is probably mediated by modulating local pathological processes, including synovial hyperplasia, leukocyte recruitment and tissue invasion, rather than by suppressing systemic cytokine levels.

### Aggressive capacity of RA-FLSs is exacerbated in iron overload environment

**Fig. 2:**
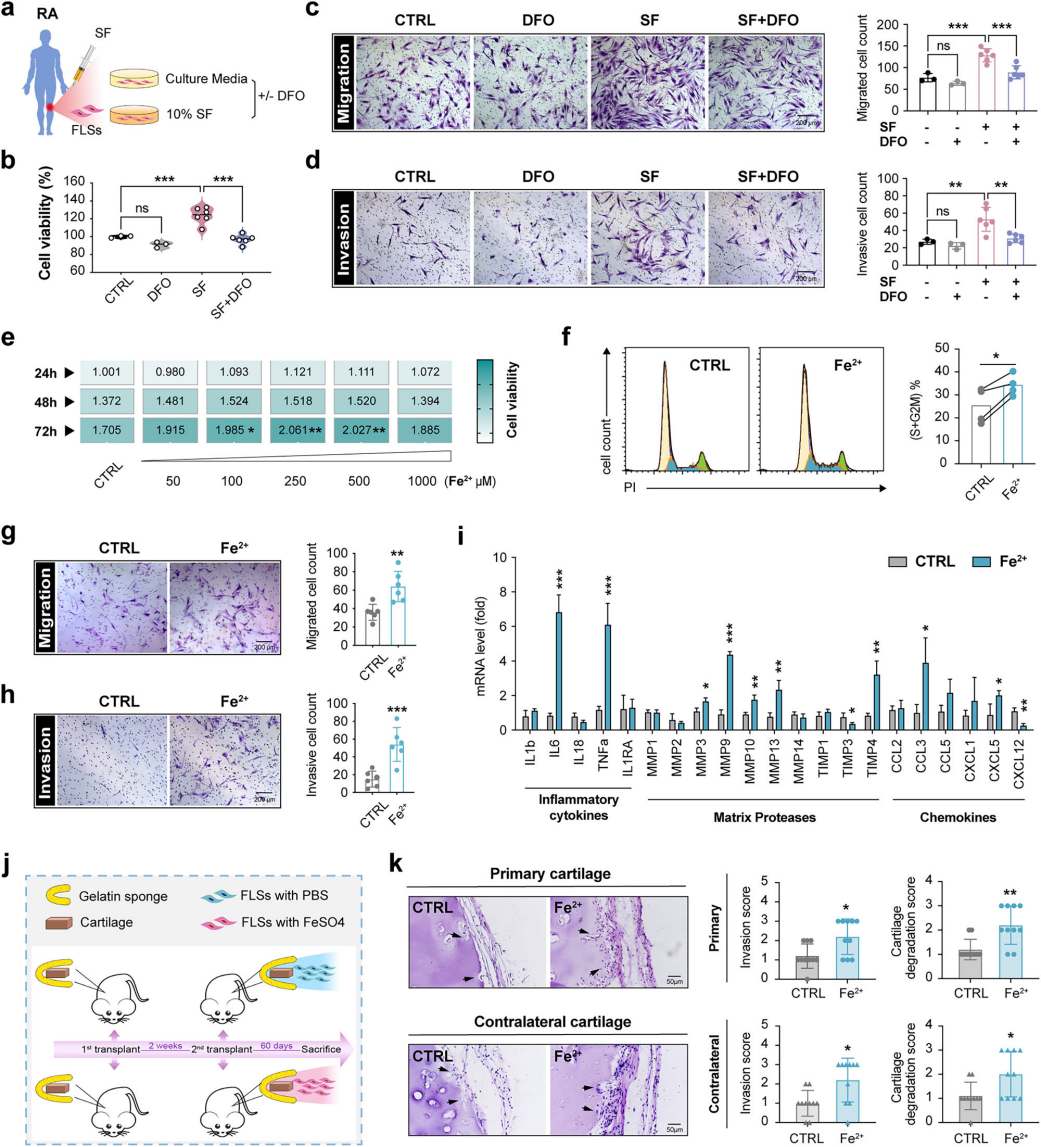
Ferrous iron promotes aggressive biology of RA-FLSs. **a** A schematic plot of the experimental process in vitro. FLSs derived from patients with RA were activated with 10% SF, followed by treatment with DFO (100 μM, Selleck, S5742) for 24 h. **b** The cell viability was assessed by CCK-8 assay kit. *N*=3 in CTRL and DFO group, *N* = 6 in SF and SF+DFO group, ***P < 0.001, ns, not significant, by one-way ANOVA (mean ± s.d.). **c**-**d** Migration (**c**) and invasion (**d**) ability of RA-FLSs was evaluated by Transwell assay using one-way ANOVA (mean ± s.d.). *N* = 3 in control (CTRL) and DFO group, *N* = 6 in SF and SF + DFO group. ns, not significant, **P < 0.01, ***P < 0.001. **e** The 500 μM FeSO_4_ that mimics the high ferrous iron environment was used to stimulate RA-FLSs dose-dependently, and cell viability was analyzed by CCK-8 assay. *N* = 4 in each group, *P < 0.05, **P < 0.01 versus CTRL group by one-way ANOVA analysis (mean ± s.d.). **f**–**i** The cell cycle distribution was detected by flow cytometry (*N* = 4 in each group) (**f**); the ability of RA-FLSs to migrate (**g**) and invade (**h**) was evaluated by Transwell assay (*N* = 6 in each group) ; FLSs cultured in a high-iron environment were subjected to a PCR array to analyze the cytokines expression associated with inflammation, chemokines and matrix proteases in vitro (**i**). In **f**–**i** the 500 μM FeSO_4_ that was close to the average ferrous iron level in RA SF was adopted to stimulate RA-FLSs for proliferation, migration, invasion and cytokine expression. *P < 0.05, **P < 0.01, ***P < 0.001 versus PBS group in Student’s *t*-test (mean ± s.d.). Data are displayed as fold change relative to FLSs from the PBS-treated group (*N* = 3 in each group). **j** The experimental design in humanized synovitis models by engrafting cartilage and RA-FLSs into SCID mice. In the first operation, SCID mice were implanted with a cartilage–gelatin composite under the left flank skin (contralateral cartilage). After 2 weeks, 5 × 10^5^ RA-FLSs were injected into the cartilage–gelatin composite, and the implant was inserted into a subcutaneous space in the right flank (primarily cartilage). The implanted RA-FLSs were immersed in the high-iron environment or CTRL vector. At day 60, the contralateral and primarily cartilages were collected. **k** The cartilage destructive lesion caused by RA-FLSs in vivo in humanized synovitis models are indicated by black arrows (*N* = 5 in each group, and two different area from each sample were collected for statistics). Data are mean ± s.d. and were analyzed using in Student’s *t*-test. *P < 0.05, ** *P* < 0.01 versus vehicle group.

FLSs are pivotal cellular mediators during the inflammation and bone destruction progression of patients with RA, exhibiting ‘tumor-like’ biology with excessive proliferation, migration and invasion. Considering the crucial role of the arthritic microenvironment, we simulated FLSs with 10% RA SF and evaluated the impact of iron by adding iron chelator DFO into the culture media (Fig. 2a). With CCK-8 assays, we found a significant enhancement in FLSs viability upon SF stimulation (Fig. 2b). In the meantime, RA SF positively regulated the migratory and invasive capabilities of FLSs using Transwell assessment (Fig. 2c,d), conforming to the reported research. The ablation of overloading iron with DFO abolished the above promotion effects, indicating that iron was an important factor in SF to induce the aggressive characteristics of FLSs in RA (Fig. 2b–d).

As most of the elevated iron in SF is in ferrous state, FeSO_4_ was used to stimulate FLSs directly in the following experiment. Unlike our previously observed ferroptosis on macrophages^9^ and reduced survival of monocytes in peripheral blood mononuclear cells of patients with RA (Supplementary Fig. 6), 500 μM FeSO_4_ that reflects the moderate concentration of ferrous iron in RA SF, did not cause significant cell death but conferred an anti-lipid peroxidation capacity on RA-FLSs (Supplementary Fig. 7a–c). Furthermore, FeSO_4_ notably enhanced FLSs’ cellular viability (Fig. 2e), induced a higher proportion of cells at the S + G2/M phase indicative of high proliferation (Fig. 2f) and increased the number of migrated and invaded cells in Transwell assays (Fig. 2g–h). In addition, during the iron-activating treatment of FLSs, matrix proteases MMP3, MMP9, MMP10 and MMP13 as well as chemokines CCL3 and CXCL5 were dramatically elevated (Fig. 2i). Moreover, the inflammatory cytokines IL-6 and TNF-α were found to be increased in the FLS response to iron. Considering the unaffected inflammatory cytokines in the paw tissues of STIA models with iron chelator DFO administration, the different synovial cells may exhibit a heterogeneous inflammatory response to iron stimulation.

To evaluate the pathogenic effect of ferrous iron on FLSs in vivo, we used a humanized synovitis model by implanting fresh cartilage and RA-FLSs into a SCID mouse model, as depicted in Fig. 2j. RA-FLSs treated with 500 μM FeSO_4_ exhibited increased invasion into the cartilage and led to markedly enhanced erosion of the primary cartilages. The contralaterally engrafted cartilages, which were not in direct contact with FLSs, also demonstrated notable destruction (Fig. 2k), suggesting that the overmigration and overinvasion of RA-FLSs play a pivotal role in orchestrating iron-induced joint destruction.

### Overloading iron in joint initiates lipid synthesis in RA synoviums

**Fig. 3:**
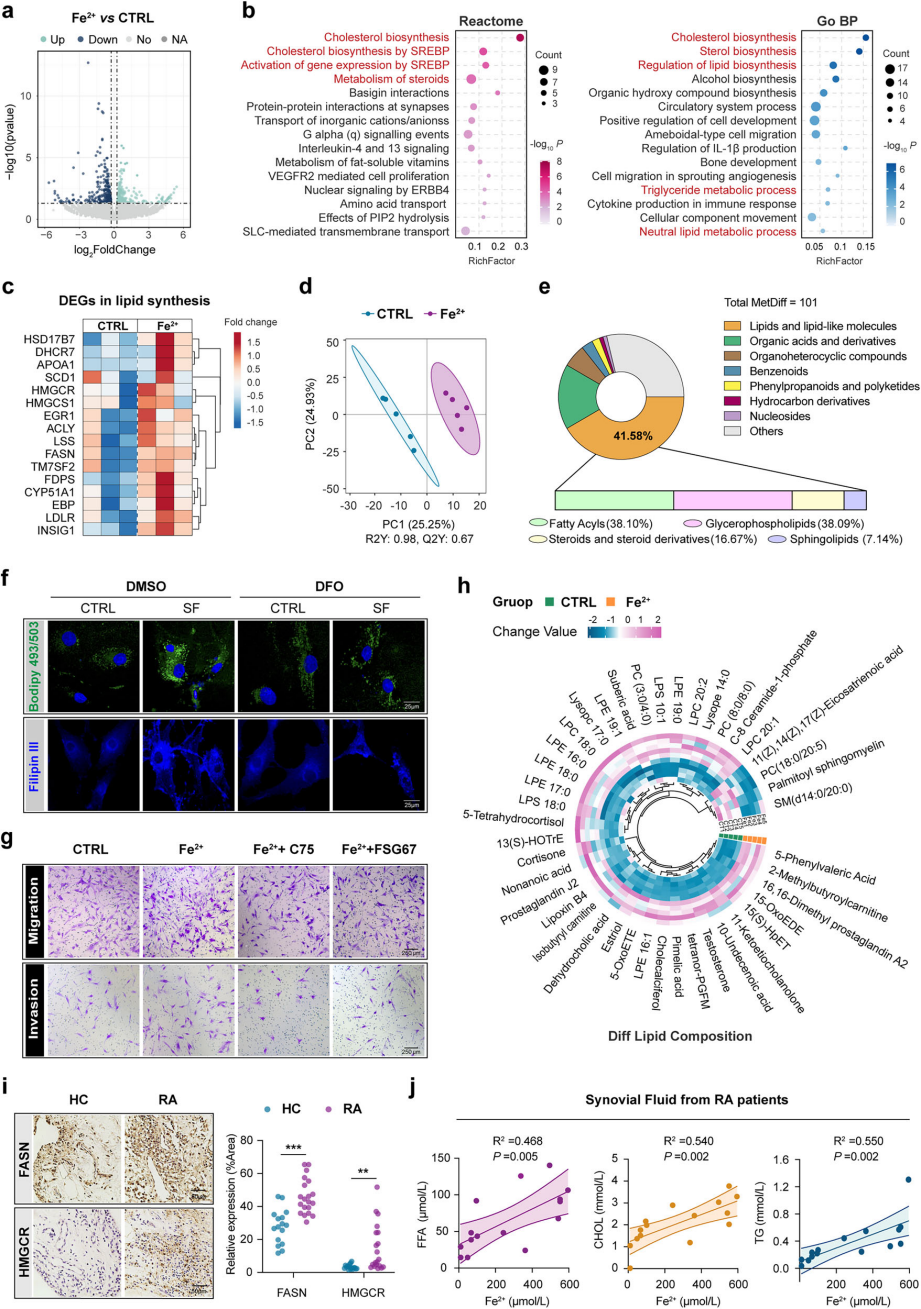
Overloading iron in joint initiates lipid synthesis in RA synoviums. **a** FLS was cultured with 500 μM FeSO_4_ for 24 h (*N* = 3), then subjected to whole transcriptome sequencing analysis; a volcano plot showing DEGs. **b** The reactome and GO enrichment for upregulated DEGs identifies lipid metabolic pathways. **c** A heat map showing FeSO_4_ upregulated several lipid synthesis-associated genes. **d** A partial least-squares discrimination analysis of RA-FLSs metabolome with FeSO_4_ and PBS treatment (*N* = 5). Each symbol represents the data of an individual sample. **e** Top: donut chart depicting the distribution of the differential metabolites by nontargeted metabolomics analysis (total of 101). Bottom: stacked graph depicting the substances composition of the differential lipids. **f** The 10% SF was used to activate FLSs, followed by DFO supplement to block the overloading iron in surrounding. Representative images of cellular neutral lipid contents stained with Bodipy 493/503 (2 μM, green) and cholesterol detected by fluorescence dye filipin III (50 μg/ml, blue) separately. **g** The effects of fatty acids and glycerophospholipids on RA-FLSs migration and invasion using the specific fatty acid synthase inhibitor C75 or glycerophospholipid synthesis inhibitor FSG67 (*N* = 3). **P < 0.01, ***P < 0.001 compared with control (CTRL) group by Student’s *t*-test (mean ± s.d.). **h** A Circo heat map illustrating the relative intensity of specifically differential lipids between FLSs with FeSO_4_ and PBS administration. **i** Left: the representative immunohistochemical staining of lipid synthetase FASN and HMGCR in synovium of RA and HC. Right: the quantitative evaluation of FASN and HMGCR expression between the two groups (*N* = 10 RA; *N* = 10 NC; two different areas from each sample were collected for statistics). **P < 0.01***P < 0.001; by Student’s *t*-test (mean ± s.d.). **j** A correlation analysis of ferrous iron level with FFA, CHOL and TG in SF using Spearman correlation analysis (*N* = 15).

To further elucidate how iron affects RA-FLSs in driving bone erosion, we analyzed the gene expression changes of FLSs with 500 μM FeSO_4_ treatment by mRNA sequencing. As shown in Fig. 3a, 293 genes were upregulated, and 315 genes were downregulated. Among the increased genes, multiple cholesterol and steroids metabolism-associated pathways/processes were significantly enriched in both reactome and Gene Ontology (GO) analysis (Fig. 3b). Several lipogenic enzymes including HSD17B7, DHCR7, APOA1, FDPS, SCD1, FASN, ACLY, LSS, CYP51A1, HMGCR, HMGCS1 and INSIG1 in RA-FLSs were markedly upregulated with FeSO_4_ treatment (Fig. 3c). In accordance with this, the intracellular levels of FFA, TG and cholesterol in RA-FLSs were consistently elevated under conditions of iron overload (Supplementary Fig. 8). Next, we used an untargeted metabolomics approach to investigate the metabolic alterations in FLSs induced by iron overload. The partial least-squares discrimination analysis revealed a significant reprogramming of the FLSs metabolomic profile (Fig. 3d). Among the 101 differentially expressed metabolites, lipids and lipid-like molecules were predominantly altered, comprising fatty acyls (38.10%), glycerophospholipids (38.09%), steroids and steroid derivatives (16.67%) and sphingolipids (7.14%) (Fig. 3e).

To further determine whether the observed lipid accumulation is a direct result caused by iron or a correlative event, we used DFO to chelate excess iron in RA SF and found effectively reversed effects of elevated neutral lipids and cholesterol, as shown by BODIPY 493/503 and filipin III staining, respectively (Fig. 3f). We then directly tested the functional relevance of iron-mediated lipid accumulation by performing targeted inhibition experiments against two dominant lipid classes. The inhibition of fatty acid synthesis (using C75) or glycerophospholipid synthesis (using FSG67) markedly attenuated the enhanced migration and Matrigel invasion capacity of iron-loaded FLSs (Fig. 3g). Together, these results indicate that iron acts as a functional driver of lipid accumulation, which in turn contributes to the pathogenic behavior of FLSs. Building on these findings, we further characterized the specific lipid species in Fig. 3h with the obvious upregulation of key bioactive lipids such as LysoPE 14:0, Tetranor-PGFM, prostaglandin J2 and LPE 16:1. Notably, the identification of these specific upregulated lipids provides potential targets for future metabolic intervention strategies aimed at mitigating FLS-driven pathology.

In addition to the elevated lipid metabolites, the obviously higher expression of lipid synthesis markers including FASN and HMGCR were detected in the synoviums of patients with RA than in those of HC patients (Fig. 3i). Furthermore, the severity of steatosis, represented by the concentrations of FFA, TG and cholesterol, showed a strongly positive correlation with ferrous iron levels in RA SF (Fig. 3j). These data confirmed a mechanistic link between lipid accumulation and iron overload in the hyperplastic synovium and SF of patients with RA, suggesting that excess iron appears to drive lipid metabolic activity in FLSs.

### Ferrous iron initiates SREBP1 signaling to enhance the de novo synthesis of lipids in RA-FLSs

**Fig. 4:**
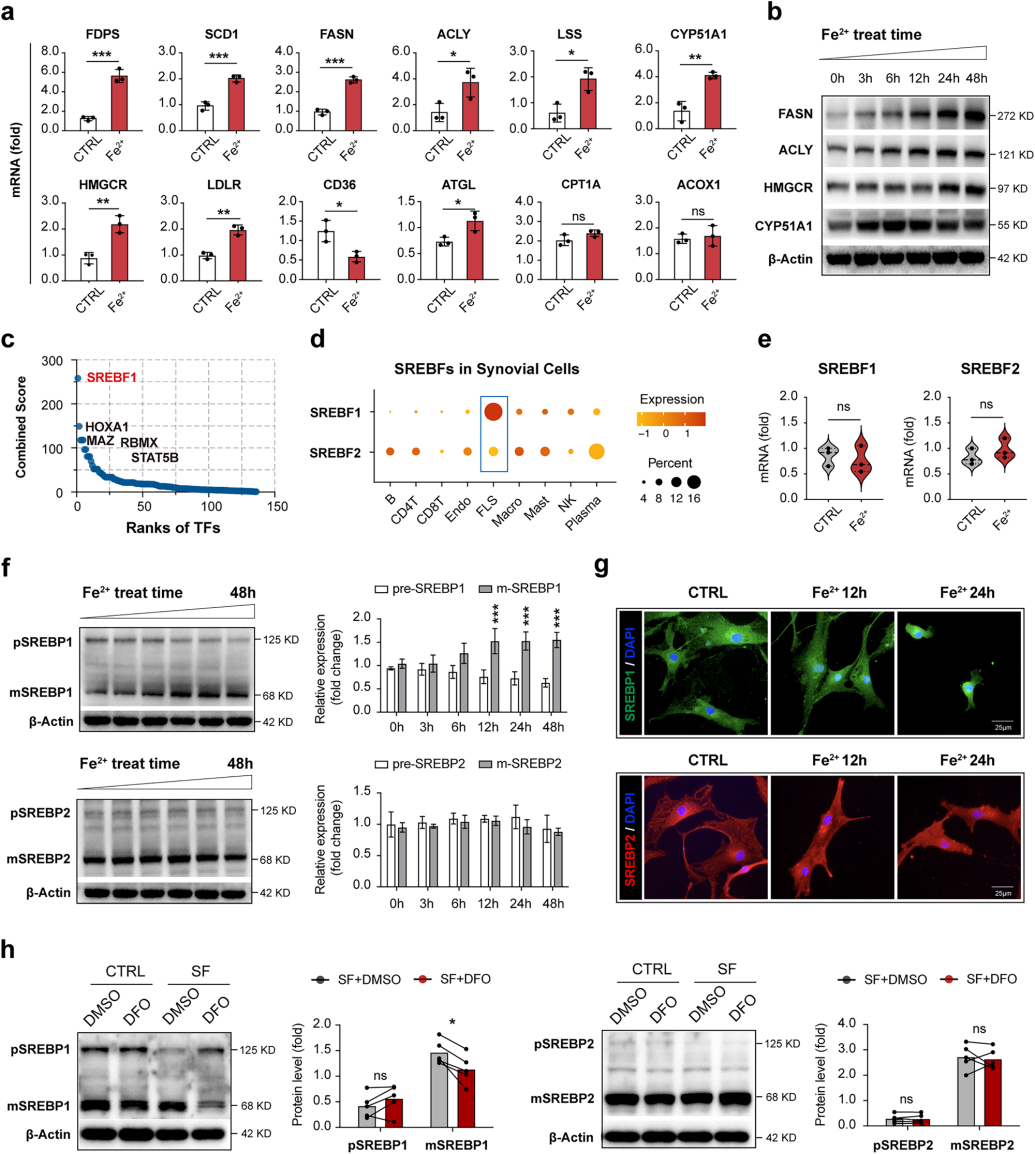
Ferrous iron initiates SREBP1 signaling to enhance the de novo lipid synthesis in RA-FLSs. **a** The expression of lipoidase genes in FLSs after 500 μM FeSO_4_ cultivation 24 h was verified with qPCR assay. Data were compared with the PBS group after normalization with β-actin mRNA level. *N* = 3, *P < 0.05, **P < 0.01, ***P < 0.001, ns, not significant, by Student’s *t*-test (mean ± s.d.). **b** The expression of lipid synthetase in RA-FLSs upon FeSO_4_ treatment for indicated time period was assessed by western blot. Data shown are representative of three biologically independent experiments. **c** The predicted transcription factors (TFs) for FeSO_4_-upregulated genes by querying TRRUST database via Enrichr. SREBF1 ranks the highest. **d** The single-cell transcriptome analysis revealed the expression distribution of SREBF1 and SREBF2 in various synovial cells. **e** The quantification of SREBF1 and SREBF2 mRNA by qPCR assay. ns, not significant, compared with PBS group; by Student’s *t*-test (*N* = 3). **f** The SREBP1 and SREBP2 expression in FLSs upon FeSO_4_ treatment for the indicated time period (500 μM) was assessed by western blot. Data are presented as mean ± s.d. with the indicated significance (compared with the 0 h group by one-way ANOVA followed by Tukey’s post-test), *N* = 3. **g** SREBP1 (green) and SREBP2 (red) upon FeSO_4_ stimulation (500 μM) for the indicated time period was evaluated with IF, and the blue (DAPI) indicates the nucleus. *N* = 3. **h** FLSs were stimulated with SF (10% in culture medium) for 24 h. The specific iron chelator DFO (100 μM) was applied to block iron in the SF. The expression of SREBP1 and SREBP2 in activated FLSs was assessed with western blot. Data were compared between SF and SF + DFO groups after normalization with the β-actin protein level in each sample. *N* = 5, *P < 0.05, ns, not significant, by paired *t*-test (mean ± s.d.).

The mechanism underlying intracellular lipid accumulation is associated with aberrant lipid intake, synthesis and metabolic processes involving oxidation and hydrolysis. The RNA-seq analysis corroborated by qPCR assays revealed that ferrous iron upregulated the mRNA levels of key lipogenic genes in FLSs, including FDPS, SCD1, FASN, ACLY, LSS, CYP51A1 and HMGCR. Despite an increase of the cholesterol uptake gene LDLR (Fig. 4a and Supplementary Fig. 9), there was no expression change of the fatty acid transporter gene CD36 (Fig. 4a and Supplementary Fig. 10). The TG hydrolase ATGL mRNA was enhanced, whereas the mRNA of genes regulating fatty acid β-oxidation, CPT1A and ACOX1 remained unchanged following iron treatment (Fig. 4a). Western blot analysis further demonstrated a time-dependent increase in the expression of de novo lipogenesis enzymes FASN, ACLY, HMGCR and CYP51A1 during iron stimulation (Fig. 4b). These results suggest that robust lipid synthesis is the predominant metabolic pathway activated by iron in FLSs.

Subsequently, to elucidate the upstream regulatory factors modulated by iron, we screened all transcription factors associated with upregulated genes in FLSs and identified SREBF1, HOXA1, MAZ, RBMX and STAT5B as the top candidates (Fig. 4c). SREBF1, which ranked the highest, encodes the sterol regulatory element-binding protein SREBP1, a master transcription factor primarily responsible for the biosynthesis of fatty acids and cholesterol. The single-cell analysis revealed a particularly high expression of SREBF1 in FLSs, whereas SREBF2, the other SREBF isoform, exhibited broad but low expression across plasma cells, T cells, B cells, endothelial cells, macrophages, NK cells and FLSs in RA synovial tissues (Fig. 4d). Although qPCR assays detected no changes in mRNA levels of SREBF1 and SREBF2 in FeSO_4_-treated FLSs (Fig. 4e), western blot analysis indicated that iron stimulation promoted the cleavage and activation of SREBP1, with a gradual decrease in the SREBP1 precursor (pSREBP1) and an increase in the mature form (mSREBP1) (Fig. 4f). Compared with unstimulated FLSs, FeSO_4_-treated cells showed a marked and time-dependent translocation of SREBP1 from the cytoplasm to the nucleus, where it acts as a transcription factor (Fig. 4g and Supplementary Fig. 11). All these changes were not observed for SREBP2 (Fig. 4f,g). Moreover, the cleavage and activation of SREBP1, rather than SREBP2, in RA-FLSs were further augmented by SF stimulation, an effect significantly reversed by the iron chelator DFO (Fig. 4h). Consistently, upregulation effects of lipid synthase caused by SF was abolished with iron chelation (Supplementary Fig. 12). Collectively, these findings imply that iron is an essential inducer of SREBP1-mediated lipogenesis in RA-FLSs.

### RA-FLSs aggression promoted by ferrous iron is dependent on SREBP1 signaling

**Fig. 5:**
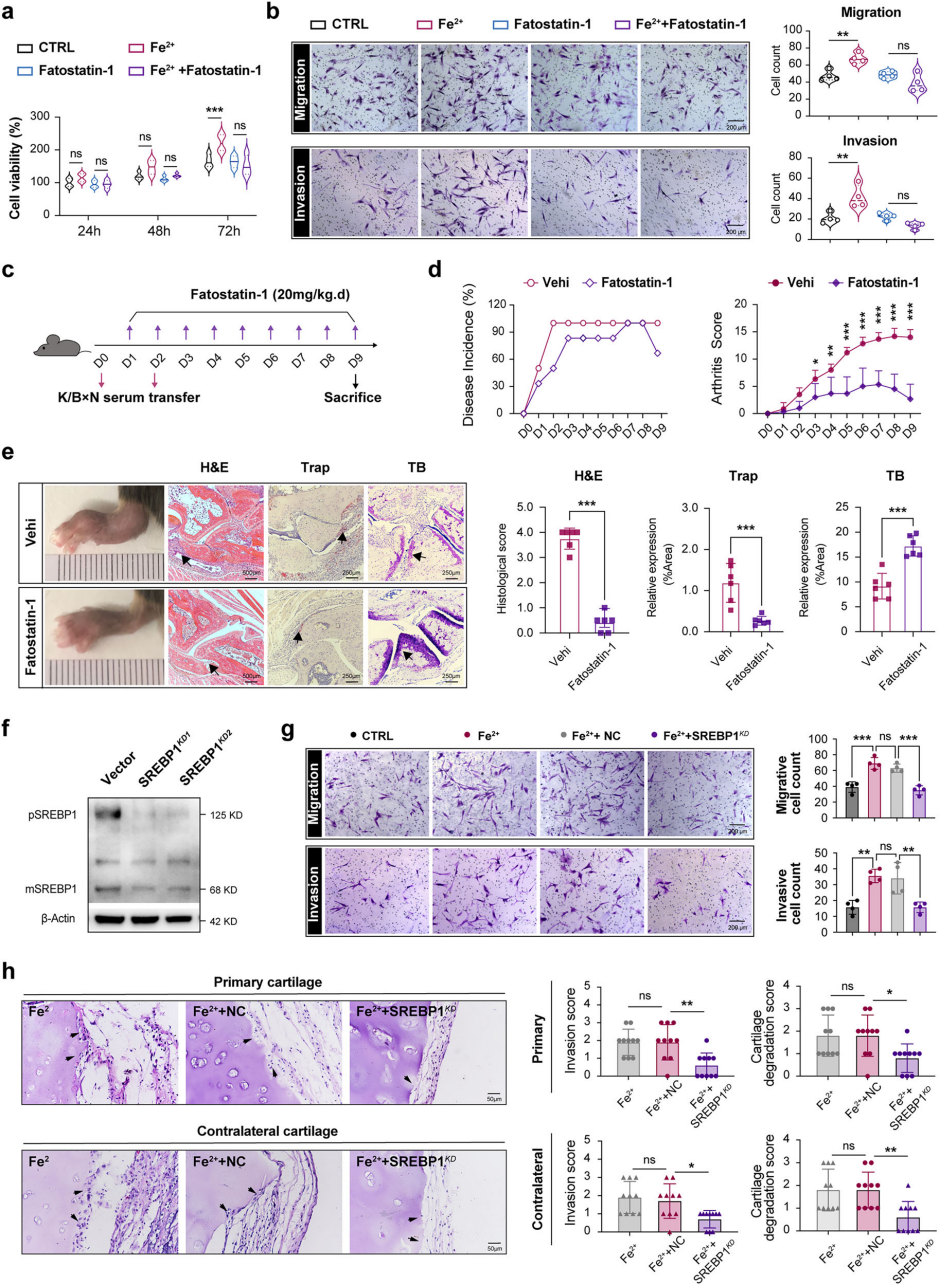
The aggressive capacity of RA-FLSs promoted by excess iron is dependent on SREBP1 signaling. **a**,**b** RA-FLS were incubated with FeSO_4_ (500 μM) alone or in combination with SREBPs-pathway inhibitor fatostatin 1 (10 μM) 24 h before the detection of cell viability, migration and invasion: cell viability was measured by CCK-8 assay (**a**), and the ability of RA-FLSs to migrate and invade was evaluated by Transwell assay (**b**). *N* = 4. **P < 0.01, ***P < 0.001, ns, not significant. Data are presented as mean ± s.d. and analyzed using one-way ANOVA. **c** A schematic diagram of fatostatin 1 treatment in the STIA model. **d** The dynamic change of disease incidence and arthritis scores were determined daily in STIA with fatostatin 1 administration and statistically analyzed at 9 days. *N* = 6 in each group. *P < 0.05, **P < 0.01, ***P < 0.001 versus vehicle (Vehi) group by Student’s *t*-test (mean ± s.d.). **e** Representative images and pathological staining section of rear paws in the model. The black arrowhead indicates proliferation of synoviocytes in H&E, osteoclast in Trap and destructive cartilage in TB. Right: statistical results. *N* = 6 in each group. Data are presented as mean ± s.d. ***P < 0.001 versus Vehi group in Student’s *t*-test. **f** RA-FLSs were infected with lentiviral vectors carrying SREBP1-shRNA or control lentivirus carrying GFP only (NC). SREBP1 expression was analyzed by western blot at 5 days after infection. Experiments were repeated in three independent individuals. **g** Transwell chemotaxis assay showed the migration and invasion change of FLSs with SREBP1 knockdown (SREBP1^KD^) upon FeSO_4_ treatment. *N* = 4. Data are mean ± s.d. and were analyzed using one-way ANOVA. **P < 0.01, ***P < 0.001, ns, not significant. **h** The migration and invasion ability in vivo of RA-FLSs with SREBP1^KD^ was evaluated in humanized synovitis models (*N* = 5 in each group, and two different area from each sample were collected for statistics). The black arrows indicated the lesions of cartilage destruction caused by RA-FLSs. Data are mean ± s.d. and were analyzed using one-way ANOVA. *P < 0.05, **P < 0.01, ns, not significant.

We next explored whether SREBP1 activation acts as a passive bystander or plays a causative role in the aggressive phenotype of RA-FLSs under iron treatment. To address this question, we used fatostatin 1, an SREBP-signaling antagonist. As shown in Fig. 5a,b, the enhanced viability, migration and erosive capacity of RA-FLSs induced by FeSO_4_ were significantly suppressed by fatostatin 1. In addition, the in vivo intervention studies using STIA model demonstrated that fatostatin 1 administration resulted in a reduced arthritis score, decreased disease incidence and a marked amelioration of pathological changes, including synovial hyperplasia, cartilage erosion and bone destruction (Fig. 5c–e).

However, it is noteworthy that fatostatin 1 functions as a nonspecific inhibitor of lipid synthesis by blocking the translocation of SREBPs from the endoplasmic reticulum (ER) to the Golgi apparatus and their subsequent activation, thereby inhibiting the cellular activation of both SREBP1 and SREBP2. To further elucidate the role of SREBP1 in modulating the iron effects on the ‘tumor-like’ characteristics of RA-FLSs, we supplemented our study with eicosapentaenoic acid, a polyunsaturated fatty acid well-established to specifically suppress the production and activation of SREBP1 without directly affecting SREBP2^17,18^ (Supplementary Fig. 13). As we expected, eicosapentaenoic acid administration recapitulated the key therapeutic effects of fatostatin 1, notably reducing the arthritis severity in the STIA model (Supplementary Fig. 14). Crucially, this SREBP1-specific pharmacological effect is directly supported by our ex vivo genetic evidence. We silenced SREBP1 expression in RA-FLSs (Fig. 5f) and observed that SREBP1 knockdown abrogated the promoting effects of excess iron on RA-FLS’s migration and invasion (Fig. 5g). In the humanized model of synovitis shown in Fig. 5h, downregulation of SREBP1 in RA-FLSs prominently abolished the promotion of invasion into both the primary and contralateral cartilages upon FeSO_4_ exposure. These findings underscore the essential role of SREBP1 in mediating the hyperproliferative and overerosive phenotypes of RA-FLSs in response to iron treatment.

### Dissociation of IRP1 from SCAP 5′ UTR mRNA activates SREBP1 and promotes FLS aggression in iron overloading environment

**Fig. 6:**
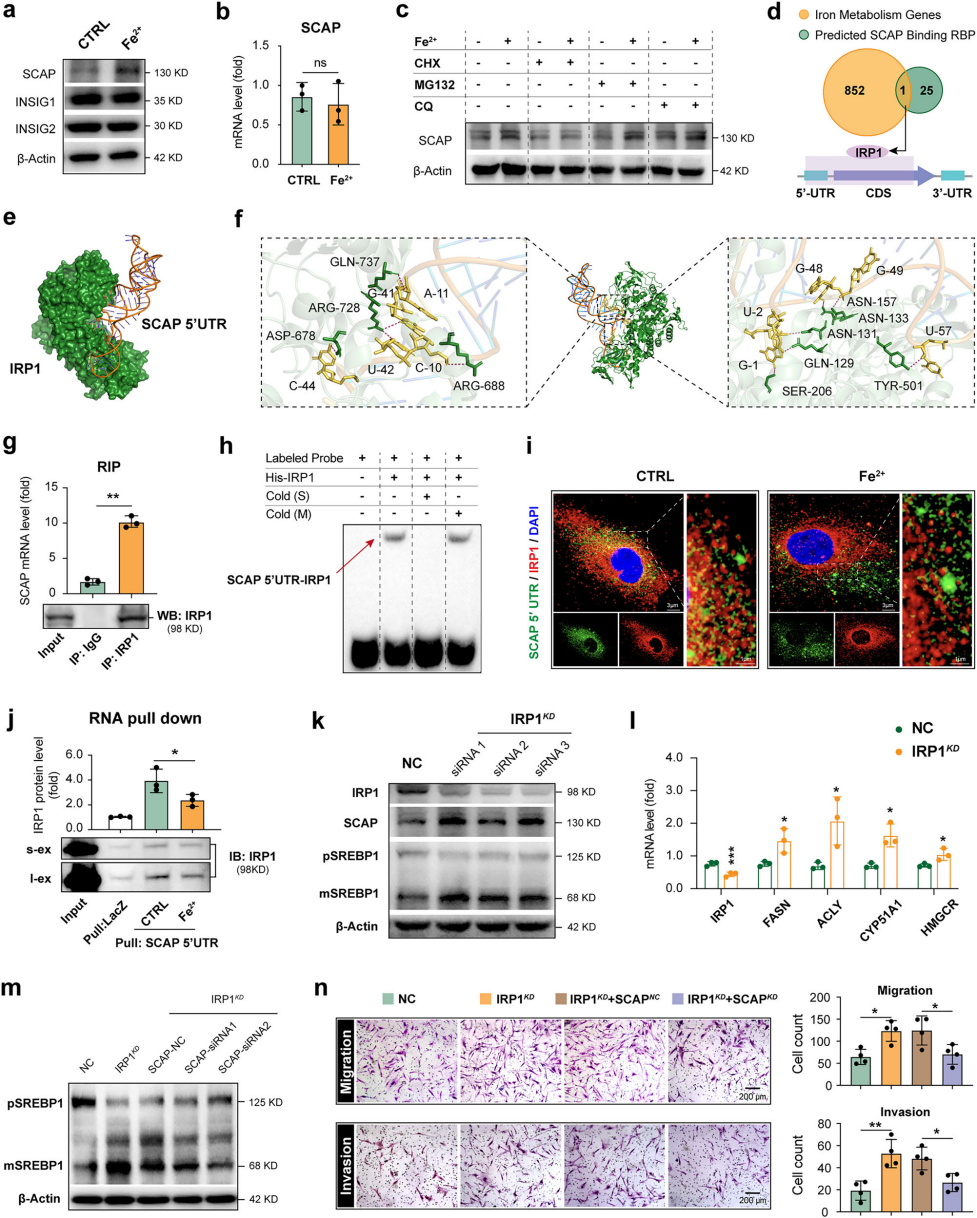
Dissociation of IRP1 from SCAP 5′ UTR mRNA activates SREBP1 and promotes FLSs aggression in iron overloading environment. **a** RA-FLSs were stimulated with FeSO_4_ (500 μM) for 3 h. The level of SCAP, INSIG1 and INSIG2 was analyzed by western blot assay. *N* = 3. **b** The SCAP mRNA expression in FLSs upon FeSO_4_ stimulation. *N* = 3. ns, not significant by Student’s *t*-test (mean ± s.d.). **c** FLSs were incubated with protein synthesis inhibitor CHX (50 μg/ml), proteasome inhibitor MG132 (10 μM) and autolysosome inhibitors CQ (10 μM) for 4 h separately to interrupt protein synthesis or degradation process accompanied with FeSO_4_ treatment. *N* = 3. **d** The cross comparison of SCAP mRNA binding proteins and iron metabolic genes focused on the only common gene IRP1 (top); the predicted binding domain of IRP1 targeting SCAP mRNA is shown (bottom). **e** A cartoon structure of the binding IRP1 and SCAP 5′ UTR complex. **f** The molecular docking for predicting interaction site between IRP1 and SCAP 5′ UTR. **g** Top: the RIP and quantitative rtPCR assays demonstrated specific binding of SCAP mRNA and IRP1. Bottom: immunoprecipitation (IP) efficiency of IRP1 antibody shown by western blot. *N* = 3. **h** The EMSA to verify the interaction between purified human recombinant IRP1 protein and the SCAP 5′ UTR probe. Cold(S), unlabeled specific competitor probe; Cold(M), unlabeled mutant competitor probe. **i** Representative FISH–IF images identified the colocalization of SCAP 5′ UTR and IRP1 in FLS cytoplasm, which was obviously reduced after FeSO_4_ treatment. The green (FAM-labeled probe) indicates the SCAP 5′ UTR, the red indicates the IRP1 protein and the blue (DAPI) indicates the nucleus. **j** The RNA pulldown assay showed the interaction between SCAP 5′ UTR and IRP1 in RA-FLSs. FeSO_4_ stimulation decreased IRP1 level pulled by SCAP 5′ UTR (s-ex, short exposure; l-ex, long exposure). *N* = 3. **k** The western blot showed the SCAP expression and subsequent SREBP1 cleavage activation in FLSs with IRP1 knockdown. For the siRNA knockdown of IRP1, FLSs were transfected with three designed siIRP1 using JetPRIME (Polyplus-transfection, 101000046). *N* = 3. **l** The relative mRNA levels of lipase genes FASN, ACLY, CYP51A1 and HMGCR in indicated FLSs with IRP knockdown detected by qPCR. *N* = 3. *P < 0.05 versus NC-transfected group in Student’s *t*-test (mean ± s.d.). **m**,**n** The concomitant SCAP knockdown rescued the elevated activation of SREBP1 (**m**), as well as impairing the enhanced migration and invasion of IRP1-deficient RA-FLSs in Transwell assays (**n**). Data are presented as mean ± s.d. *P < 0.05, ** *P* < 0.01 versus NC-transfected group in one-way ANOVA. MG132, carbobenzoxy-L-leucyl–L-leucyl-L-leucinal; 3-MA, 3-methyladenine.

During the process of lipids synthesis, SCAP plays a pivotal role in facilitating the transport of SREBPs from the ER to the Golgi apparatus and their following cleavage activation. Insulin-induced genes (INSIGs), including INSIG1 and INSIG2, serve as the primary negative regulators of SREBPs–SCAP transport and proteolytic activation. Our data revealed that treatment RA-FLSs with FeSO_4_ led to a rapid increase in SCAP protein expression as early as 3 h with subsequently time-dependent effect (Fig. 6a and Supplementary Fig. 15), whereas there was no significant alternation detected in the levels of INSIG1 and INSIG2. Interestingly, qPCR results showed unchanged levels of SCAP mRNA (Fig. 6b), suggesting that the elevated level of SCAP may be attributed to enhanced protein synthesis or damaged degradation processes. Further treatment of FLSs with the protein synthesis inhibitor cycloheximide (CHX) effectively abrogated FeSO_4_-induced upregulation of SCAP, whereas proteasome inhibitor MG132 and autophagy lysosome inhibitors chloroquine (CQ) did not exhibit any significant effects on SCAP expression (Fig. 6c). These findings indicate that the elevated level of SCAP expression in RA-FLSs following FeSO_4_ treatment is probably due to enhanced translation rather than a reduction in protein degradation.

The sequence of 5′ UTR or 3′ UTR in mRNA is a critical determinant of translation efficiency and can be modulated by RNA-binding protein occupancy. In view of this, we intersected the SCAP targeted RNA-binding proteins that identified in the RBPDB database (the database of RNA-binding protein specificities, http://rbpdb.ccbr.utoronto.ca/index.php), with iron metabolism genes listed in the GeneCards database (https://www.genecards.org). Our analysis revealed that IRP1 was the only shared gene, potentially occupying both the 5′ UTR and coding sequence (CDS) regions of SCAP mRNA (Fig. 6d). This led us to hypothesize that IRP1 binding may be a key regulatory step in SCAP translation. Next, molecular docking simulations predicted the distribution and binding sites of SCAP 5′ UTR mRNA and IRP1 protein (Fig. 6e,f). RIP assays confirmed the endogenous interaction between IRP1 and the SCAP 5′ UTR under physiological conditions (Fig. 6g). To determine if this interaction is direct, we performed EMSA using the SCAP 5′ UTR RNA probe and purified recombinant human IRP1 protein. A distinct mobility shift was observed upon incubation of the probe with HIS-IRP1 (Fig. 6h, lane 2), which was specifically competed by a 50-fold molar excess of unlabeled wild-type probe (Fig. 6h, lane 3) but not by a mutant probe (Fig. 6h, lane 4). Furthermore, FISH–IF analysis showed that iron overload (FeSO₄ stimulation) induced the dissociation of IRP1 from the SCAP 5′ UTR in cells (Fig. 6i). Consistently, RNA pulldown assays using lysates from FeSO₄-treated FLSs revealed a marked reduction in IRP1 binding to the SCAP 5′ UTR compared with controls (Fig. 6j). Taken together, these results demonstrate that IRP1 directly and specifically binds the SCAP 5′ UTR, and this interaction is regulated in a iron-dependent manner.

A previous study has reported that IRP1 and IRP2 both function as the iron-responsive element–RNA-binding proteins. When iron is sufficient, IRP1 shifted from the RNA-binding protein to aconitase^19^. Therefore, we performed IRP1 knockdown using siRNA to mimic the loss of RNA-binding function and assessed its impact on SCAP expression and SREBP1 activation in RA-FLSs. Compared with the NC group, the knockdown of IRP1 led to increased SCAP expression and enhanced cleavage of SREBP1, in contrast to the unaltered expression after IRP2 interference (Fig. 6k and Supplementary Fig. 16). Moreover, an upregulating transcription of SREBP1 target genes, including FASN, ACLY, CYP51A1 and HMGCR, was observed in IRP1-knockdown FLSs (Fig. 6l). To validate SCAP as the essential downstream mediator of IRP1, we conducted a rescue experiment in which SCAP was knocked down in IRP1-deficient FLS cells. Strikingly, SCAP knockdown effectively reversed the upregulation of mSREBP1 caused by IRP1 silencing (Fig. 6m). Furthermore, the enhanced migratory and invasive capacities resulting from IRP1 depletion were significantly suppressed by simultaneous SCAP knockdown (Fig. 6n). These findings demonstrate that SCAP acts as a critical downstream effector through which IRP1 regulates SREBP1 processing, thereby promoting the aggressive phenotype of RA-FLSs under conditions of iron overload. Nonetheless, given the shared IRE-binding functions between IRP1 and IRP2, we investigated the possibility of direct compensatory upregulation and found that IRP2 protein levels were not elevated in IRP1-knockdown FLSs (Supplementary Fig. 17). This suggests that significant immediate compensation by IRP2 may not occur within our experimental model and timeframe; however, more complex forms of synergy or context-dependent mutual influence between the two molecular constitute a valuable direction for future research.

## Discussion

The current study elucidates the adverse impact of excess iron in arthritic microenvironment on the functional impairments and disability outcomes in patients with RA. Our findings demonstrate that the elevated levels of ferrous iron in the joint milieu exacerbate synovial hyperplasia and bone destruction in RA, as assessed by the MRI. Mechanistically, the iron regulatory protein IRP1 emerges as a critical mediator to orchestrate the cleavage activation of the transcription factor SREBP1 via interplaying with the upstream adaptor protein SCAP. This interaction redirects the intracellular lipid metabolism and endows RA-FLSs with enhanced proliferative and erosive ability, thereby contributing to joint damage and disease progress.

Iron homeostasis is a prerequisite for maintaining essential physiological functions, including oxygen transport, cellular energy metabolism, electron transport and a myriad of enzymatic reactions^20^. As stated by the World Health Organization, iron deficiency and its consequent anemia are among the most prevalent public health challenges^21^. In the context of numerous chronic conditions, systemic iron deficiency is often paralleled by anomalous iron accumulation at the site of pathology, such as in degenerative diseases (for example, Alzheimer’s disease and Parkinson’s)^22,23^, chronic inflammatory disease (for example, atherosclerosis)^24^, metabolic disorders (for example, obesity and liver lipogenesis)^8,25^ and autoimmune diseases (for example, multiple sclerosis)^26^. Local iron overload is also a feature of the microenvironment in the RA joint, which probably originates from dysregulated iron handling by tissue-resident and infiltrating immune cells. Macrophages, as master regulators of iron homeostasis, are a prime candidate^27,28^. In the inflamed joint, synovial macrophages may contribute to local iron accumulation through repeated phagocytosis of erythrocytes from micro-bleeds and through inflammation-induced suppression of iron export (for example, via hepcidin signaling), potentially leading to the aberrant release of labile iron into the synovial microenvironment^29^. Dysregulated iron metabolism contributes to an escalated production of oxygen-based radicals, inflicting damage upon multiple cells and tissues. In RA, we have previously demonstrated that iron exacerbates focal immune dysregulation within the joint by inducing ferroptosis in anti-inflammatory macrophages, while sparing proinflammatory counterparts^9^. In this study, we observed that ferrous iron bolsters cellular anti-lipid peroxidation and endows RA-FLSs with resistance to ferroptosis. This protective effect may be associated with the concurrent activation of the GPX4 antioxidant system that has been reported alongside dysregulated lipid metabolism^30^. Building on this survival advantage, ferrous iron indeed endows RA-FLSs with greatly aggressive properties, such as enhanced migration and invasion. Given the pivotal role of FLSs in RA bone erosion, we hypothesize that the destructive effects of iron-treated FLSs are more pronounced, as corroborated by humanized synovitis models. Established evidence suggests that although iron deficiency anemia is a common comorbidity in RA, systemic iron supplementation should be approached with caution as it has been reported to exacerbate synovitis^31^. Considering the distinct responses of synovial cells to excess iron, targeted iron interventions specific to certain cell types may offer a more effective therapeutic strategy for RA management.

Synovial hyperplasia and invasiveness are processes dependent on abundant energy reserves. Lipids serve as crucial nutrients, providing both the energy and the building blocks necessary to fuel uncontrolled cellular proliferation and disease progression in various pathologies, including hepatocellular carcinoma^32^, ovarian cancer^33^ and glioblastoma^34^. As early as 1962, Bole et al. reported increased levels of phospholipids, cholesterol and neutral lipids in the SF of patients with RA^35^. Intracellular fatty acids and lipid droplets have also been implicated in the tissue-invasive properties of RA T cells^36^. In addition, metabolite profiling analysis using gas chromatography–time-of-flight mass spectrometry revealed significantly elevated fatty acid metabolism alongside severely disrupted glucose metabolism in RA-FLSs^37^. Recent research has demonstrated that RA-FLSs are more reliant on fatty acid metabolism compared with glucose and amino acids^38^. SREBPs, comprising two isoforms, are central transcription factors in lipid synthesis. SREBP1 acts as a potent activator of downstream lipogenic enzymes involved in the synthesis of cholesterol, fatty acid and TG, whereas SREBP2 preferentially enhances the transcription of genes required for cholesterol synthesis^39^. In this study, through a combined transcriptomic and metabolomic analysis, we have brought considerable attention to the heightened lipid metabolism observed in iron-treated RA-FLSs and identified SREBP1, rather than SREBP2, as the central hub of this metabolic activity. Notably, our data also showed the upregulation of LDLR, a receptor mediating the cellular uptake of cholesterol-rich low-density lipoprotein particles in response to iron, suggests that iron overload may promote lipid accumulation in RA-FLSs not only by enhancing de novo lipogenesis but also involving the exogenous acquisition of sterols from the microenvironment. Furthermore, the remodeled lipid metabolism supplies the necessary energetic building blocks and signaling cues to sustain excessive cytoskeletal reorganization, membrane protrusion and matrix degradation, thereby fueling the aggressive phenotype of RA-FLSs. These findings align closely with recent reports that SREBP1 positively regulates the PI3K–AKT–mTOR pathway in RA-FLSs^30^. Critically, our data establish focal iron overload as a primary driver of these metabolic disturbances. Together, these results provide novel insights into RA pathophysiology and underscore the pivotal role of the disordered arthritic microenvironment in disease progression.

The activation of SREBPs is contingent upon their translocation from the ER to the Golgi apparatus for undergoing cleavage and subsequent nuclear translocation^39^. During this process, SCAP serves as an escort protein, forming a stabilizing complex with SREBPs to facilitate their transport. Conversely, ER-resident INSIG proteins INSIG1 and INSIG2 retain SREBPs in the ER, thereby hijacking their trafficking and activation. Emerging evidence implicates a link between iron homeostasis and lipid metabolism in various pathophysiological conditions^25,40^; however, the direct crosstalk between these two processes remains elusive. SREBF2 has been shown to promote transferrin (TF) transcription by activating its promoter, whereas TF expression may indirectly influence SREBP target genes through alternative pathways^41^. In this study, we identify the IRP1–SCAP–SREBP1 axis emerges as a newly defined interplay hub for iron–lipid interactions. We demonstrate that the 5′ UTR of SCAP mRNA is occupied by the iron regulator IRP1, which not only senses environmental iron change but also functions as an RNA-binding protein. Upon exposure to excess iron, IRP1 detaches from the 5′ UTR of SCAP mRNA, thereby promoting its posttranscriptional translation and facilitating the subsequent cleavage activation of SREBP1. Moreover, scRNA-seq analysis reveals that the expression of IRP1 and SCAP is almost exclusively concentrated in FLSs, highlighting a cell-specific mechanism for iron–lipid interactions in RA. Given that indiscriminate iron intervention can inevitably disrupt essential physiological functions, targeting the IRP1–SCAP interaction offers a more promising therapeutic strategy. This approach may allow for precise inhibition of FLS-driven RA progression, while avoiding the systemic consequences of broad iron manipulation.

Although this study delineates a novel iron-responsive pathway in RA-FLSs, certain limitations highlight avenues for future investigation. First, our clinical cohort established a proof-of-concept correlation; its size is limited. Validation in larger, stratified cohorts with comprehensive metadata is required to confirm generalizability. Second, the experimental scope was confined to an acute STIA model and the humanized synovitis model in this study, using FLS-specific conditional knockout mice in chronic autoimmune settings (for example, CIA) is essential to validate the axis’s pathologic relevance within a complete immune milieu. Finally, the therapeutic potential awaits pharmacological development. A logical next step grounded in our mechanistic discovery, which involves screening small-molecule libraries or designing peptides, validating their efficacy in disrupting the IRP1–SCAP 5′ UTR complex in vitro and testing the lead compound’s ability to suppress SREBP1 activation and FLS invasion in cellular and animal models, would be crucial for developing novel RA therapeutics.

In conclusion, our study provides compelling evidence that iron accumulation within the arthritic microenvironment is a key factor to exacerbate RA joint destruction. Ferrous iron positively regulates lipid metabolism through the interaction between the iron regulatory protein IRP1 and lipids synthesis mediator SCAP and subsequently enhances the aggressive phenotype of FLSs. Targeting this cell-intrinsic iron–lipid nexus in FLSs emerges as a coherent and promising therapeutic strategy to mitigate joint destruction in RA.

## Supplementary information

Supplementary Information

## Data Availability

All data needed to evaluate the conclusions are are available within the Article and its Supplementary Information. The datasets/databases used in the study along with appropriately accessible links. RNA-seq and liquid chromatography–mass spectrometry data analyzed during the current study are available from the corresponding author on reasonable request
