# Supplementary material for: Iron overload reprogramming lipid metabolism through the IRP1–SCAP axis in fibroblast-like synoviocytes aggravates bone destruction in rheumatoid arthritis
Source: Exp Mol Med. 2026 May 1;58(5):1479–94. doi: 10.1038/s12276-026-01710-6 (PMC13234328; doi:10.1038/s12276-026-01710-6)
Supplement: Supplementary file 1 — Supplementary Information [file 12276_2026_1710_MOESM1_ESM.pdf]

Supplementary materials

Supplementary Tables

Supplementary Table 1. Clinical characteristics of RA, OA, GA individuals and RA treatment history for iron content analysis in synovial fluid.

Clinical characteristics	RA (N=15)	OA (N=8)	GA (N=8)
Age (year)	56.20 ± 10.62	59.88 ± 7.04	51.25 ± 8.58
Female sex, (%)	66.67	62.50	12.50
Disease duration, year	12.20 ± 9.41	17.75 ± 8.01	4.88 ± 3.18
CRP (mg/dl)	21.73 ± 30.49	24.50 ± 20.47	25.00 ± 15.66
ESR (mm/hour)	55.60 ± 39.18	33.88 ± 18.90	45.75 ± 23.11
DAS28	4.59 ± 1.64	na	na
Prednisone Acetate, (%)	53.30	na	na
NSAIDs, (%)	80.00	na	na
Methotrexate, (%)	86.67	na	na
Leflunomide, (%)	13.33	na	na
Iguratimod, (%)	33.33	na	na
TNFi, (%)	13.33	na	na
IL-6Ri, (%)	6.70	na	na
JAKi, (%)	26.67	na	na

Results were presented as mean ± standard deviation (SD). RA, rheumatoid arthritis; OA, osteoarthritis; GA, gouty arthritis; CRP, C-reactive protein; ESR, erythrocyte sedimentation rate; DAS28, 28-joint disease activity score; NSAIDs, nonsteroidal anti-inflammatory drugs; na, not applicable.

Supplementary Table 2. Clinical characteristics of HC and RA individuals for immunohistochemical detection of synovial tissue

Clinical characteristics	HC (N=10)	RA (N=10)
Age (y)	44.50 ± 8.42	50.60 ± 8.22
Female sex, (%)	30	80
CRP (mg/dl)	15.20 ± 8.94	24.60 ± 33.10
ESR (mm/h)	30.00 ± 11.90	69.01± 37.31
DAS28	na	5.01 ± 7.65
Prednisone Acetate, (%)	na	40.00
NSAIDs, (%)	na	80.00
Methotrexate, (%)	na	100.00
Leflunomide, (%)	na	20.00
Iguratimod, (%)	na	30.00
TNFi, (%)	na	20.00
IL-6Ri, (%)	na	10.00
JAKi, (%)	na	30.00

Results were presented as mean ± standard deviation (SD). HC, healthy control; RA, rheumatoid arthritis; CRP, C-reactive protein; ESR, erythrocyte sedimentation rate; DAS28, 28-joint disease activity score; NSAIDs, nonsteroidal anti-inflammatory drugs; na, not applicable.

Supplementary Table 3. Clinical characteristics of RA patients for MR imaging analysis.

Clinical characteristics	RA (N=11)
Age, y	52.91± 10.13
Female sex, (%)	81.82
Disease duration, year	10.73 ± 8.62
CRP (mg/dl)	28.56± 33.26
ESR (mm/hour)	68.27 ± 36.45
DAS28	5.13 ± 1.56
Prednisone Acetate, (%)	72.73
NSAIDs, (%)	81.82
Methotrexate, (%)	72.73
Leflunomide, (%)	18.18
Iguratimod, (%)	18.18
TNFi, (%)	9.09
IL-6Ri, (%)	9.09
JAKi, (%)	27.27

Results were presented as mean ± standard deviation (SD). RA, rheumatoid arthritis; CRP, C-reactive protein; ESR, erythrocyte sedimentation rate; DAS28, 28-joint disease activity score; NSAIDs, nonsteroidal anti-inflammatory drugs; na, not applicable.

Supplementary Table 4. Clinical characteristics of RA patients for isolation of synovial fibroblasts

**Clinical characteristics**	**RA (N=6)**
Age, y	57.17± 8.84
Female sex, (%)	83.33
Disease duration, year	11.5 ± 7.61
CRP (mg/dl)	7.67 ± 3.82
ESR (mm/hour)	31.17 ± 16.24
DAS28	3.48 ± 0.83
Prednisone Acetate, (%)	33.33
NSAIDs, (%)	66.67
Methotrexate, (%)	83.33
Leflunomide, (%)	33.33
Iguratimod, (%)	33.33
TNFi, (%)	33.33
IL-6Ri, (%)	16.67
JAKi, (%)	16.67

Results were presented as mean ± standard deviation (SD). RA, rheumatoid arthritis; CRP, C-reactive protein; ESR, erythrocyte sedimentation rate; DAS28, 28-joint disease activity score.

Supplementary Table 5. PCR Primers used in the study

Species	Name	Sequence
Human	*FASN*-F	TTCTACGGCTCCACGCTCTTCC
Human	*FASN*-R	GAAGAGTCTTCGTCAGCCAGGA
Human	*ACLY*-F	GCTCTGCCTATGACAGCACCAT
Human	*ACLY*-R	GTCCGATGATGGTCACTCCCTT
Human	*SCD1*-F	CCTGGTTTCACTTGGAGCTGTG
Human	*SCD1*-R	TGTGGTGAAGTTGATGTGCCAGC
Human	*CYP51A1*-F	CTCTTACCAGGTTGGCTGCCTT
Human	*CYP51A1*-R	CTTGAGACTGTCTGCGTTTCTGG
Human	*FDPS*-F	CTTTCTTCCTGGTGGCAGATGAC
Human	*FDPS*-R	AGAGCTTCAGCAGGCGGTAGAT
Human	*LSS*-F	GACGACCGATTCACCAAGAGCA
Human	*LSS*-R	AGACATGCTCCTGGAAGGCAGT
Human	*LDLR*-F	GAATCTACTGGTCTGACCTGTCC
Human	*LDLR*-R	GGTCCAGTAGATGTTGCTGTGG
Human	*HMGCR*-F	GACGTGAACCTATGCTGGTCAG
Human	*HMGCR*-R	GGTATCTGTTTCAGCCACTAAGG
Human	CPT1A-F	GATCCTGGACAATACCTCGGAG
Human	*CPT1A*-R	CTCCACAGCATCAAGAGACTGC
Human	*ACOX1*-F	GGCGCATACATGAAGGAGACCT
Human	*ACOX1*-R	AGGTGAAAGCCTTCAGTCCAGC

Species	Name	Sequence
Human	*CD36-F*	CAGGTCAACCTATTGGTCAAGCC
Human	*CD36-R*	GCCTTCTCATCACCAATGGTCC
Human	*ATGL-F*	CCCACTTCAACTCCAAGGACGA
Human	*ATGL-R*	GCAGGTTGTCTGAAATGCCACC
Human	*SREBF1*-F	ACTTCTGGAGGCATCGCAAGCA
Human	*SREBF1*-R	AGGTTCCAGAGGAGGCTACAAG
Human	*SREBF2*-F	CTCCATTGACTCTGAGCCAGGA
Human	*SREBF2*-R	GAATCCGTGAGCGGTCTACCAT
Human	*SCAP*-F	TCACGTTGCAGCCGTCTTCCTT
Human	*SCAP*-R	CAGGATGCCAATCCAGACAACG
Human	*IL1β*-F	CCACAGACCTTCCAGGAGAATG
Human	*IL1β*-R	GTGCAGTTCAGTGATCGTACAGG
Human	IL6-F	AGACAGCCACTCACCTCTTCAG
Human	IL6-R	TTCTGCCAGTGCCTCTTTGCTG
Human	*IL18*-F	GATAGCCAGCCTAGAGGTATGG
Human	*IL18-R*	CCTTGATGTTATCAGGAGGATTCA
Human	*TNFα*-F	CTCTTCTGCCTGCTGCACTTTG
Human	*TNFα*-R	ATGGGCTACAGGCTTGTCACTC
Human	*MMP1*-F	ATGAAGCAGCCCAGATGTGGAG
Human	*MMP1-R*	TGGTCCACATCTGCTCTTGGCA

Species	Name	Sequence
Human	*MMP2*-F	AGCGAGTGGATGCCGCCTTTAA
Human	*MMP2-R*	CATTCCAGGCATCTGCGATGAG
Human	*MMP3*-F	CACTCACAGACCTGACTCGGTT
Human	*MMP3-R*	AAGCAGGATCACAGTTGGCTGG
Human	*MMP9*-F	GCCACTACTGTGCCTTTGAGTC
Human	*MMP9*-R	CCCTCAGAGAATCGCCAGTACT
Human	*MMP10*-F	TCCAGGCTGTATGAAGGAGAGG
Human	*MMP10-R*	GGTAGGCATGAGCCAAACTGTG
Human	*MMP13*-F	CCTTGATGCCATTACCAGTCTCC
Human	*MMP13*-R	AAACAGCTCCGCATCAACCTGC
Human	*MMP14*-F	CCTTGGACTGTCAGGAATGAGG
Human	*MMP14*-R	TTCTCCGTGTCCATCCACTGGT
Human	*TIMP1*-F	GGAGAGTGTCTGCGGATACTTC
Human	*TIMP1*-R	GCAGGTAGTGATGTGCAAGAGTC
Human	*TIMP3*-F	TACCGAGGCTTCACCAAGATGC
Human	*TIMP3*-R	CATCTTGCCATCATAGACGCGAC
Human	*TIMP4*-F	CACTACCATCTGAACTGTGGCTG
Human	*TIMP4*-R	GCTTTCGTTCCAACAGCCAGTC
Human	*CCL2*-F	AGAATCACCAGCAGCAAGTGTCC
Human	*CCL2*-R	TCCTGAACCCACTTCTGCTTGG

Species	Name	Sequence
Human	*CCL3*-F	ACTTTGAGACGAGCAGCCAGTG
Human	*CCL3*-R	TTTCTGGACCCACTCCTCACTG
Human	*CCL5*-F	CCTGCTGCTTTGCCTACATTGC
Human	*CCL5*-R	ACACACTTGGCGGTTCTTTCGG
Human	*CXCL1*-F	AGCTTGCCTCAATCCTGCATCC
Human	*CXCL1*-R	TCCTTCAGGAACAGCCACCAGT
Human	*CXCL5*-F	CAGACCACGCAAGGAGTTCATC
Human	*CXCL5*-R	TTCCTTCCCGTTCTTCAGGGAG
Human	*CXCL12*-F	CTCAACACTCCAAACTGTGCCC
Human	*CXCL12*-R	CTCCAGGTACTCCTGAATCCAC
Human	*β-ACTIN*-F	CACCATTGGCAATGAGCGGTTC
Human	*β-ACTIN*-R	AGGTCTTTGCGGATGTCCACGT

Supplementary Table 6. The sequences of siRNA and shRNA

Names	Sequence (5’-3’)
SREBP1 shRNA1	GCCTATTTGACCCACCCTATG
SREBP1 shRNA2	GCAAATCTCTGAAGGATCTGG
IRP1 siRNA1-sense	CAAGAACGAUACACUAUCA
IRP1 siRNA1-antisense	UGAUAGUGUAUCGUUCUUG
IRP1 siRNA2-sense	CCUGCUGCUCGCUACUUAA
IRP1 siRNA2-antisense	UUAAGUAGCGAGCAGCAGG
IRP1 siRNA3-sense	GACCGAGCUACGAUUGCUA
IRP1 siRNA3-antisense	UAGCAAUCGUAGCUCGGUC
IRP2 siRNA1-sense	GUUAUCAGUUGUACCAAUA
IRP2 siRNA1-antisense	UAUUGGUACAACUGAUAAC
IRP2 siRNA2-sense	GCCGCUAAGUAUUUGACAA
IRP2 siRNA2-antisense	UUGUCAAAUACUUAGCGGC
IRP2 siRNA3-sense	GGACUUAAAGUCUACUUAU
IRP2 siRNA3-antisense	AUAAGUAGACUUUAAGUCC
SCAP siRNA1-sense	GAGACGUCACGCUGUACAA
SCAP siRNA1-antisense	UUGUACAGCGUGACGUCUC
SCAP siRNA2-sense	GGCUGCGUGUUGUCUACUU
SCAP siRNA2-antisense	AAGUAGACAACACGCAGCC

Supplementary Figures

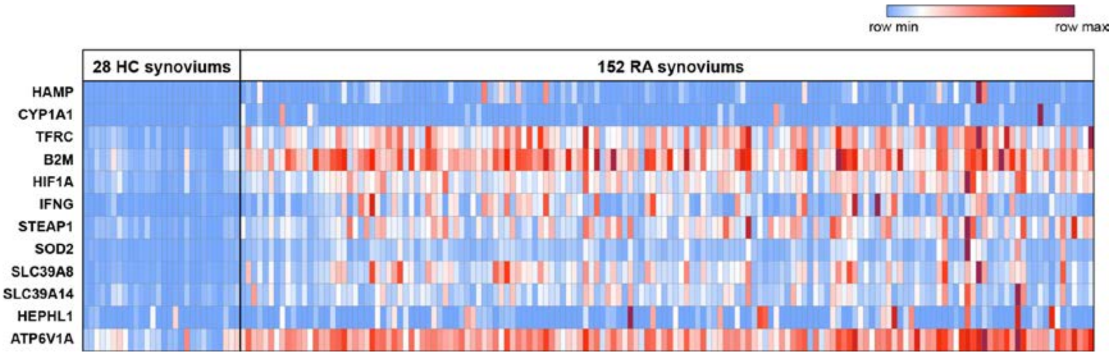

Supplementary Fig. 1. Heat map showing the different expressions of iron associated proteins in synovial tissues between 28 HC and 152 RA patients in GSE89408 datasets. HC, healthy control. RA, rheumatoid arthritis.

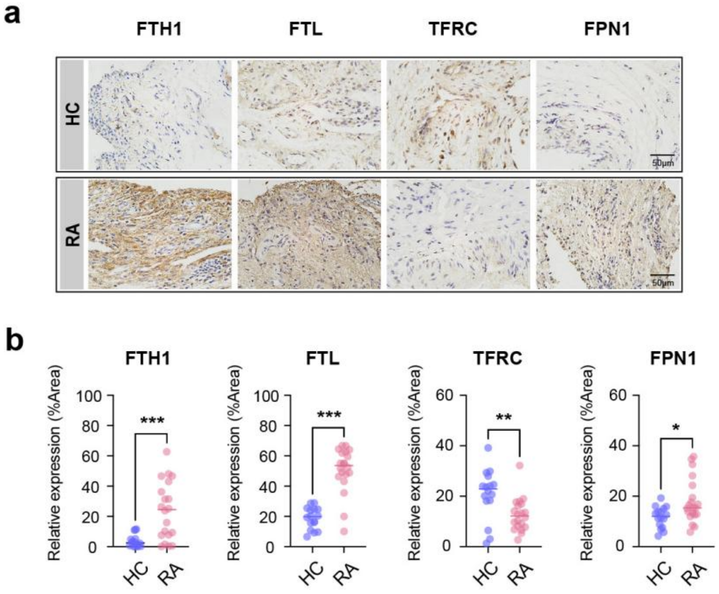

Supplementary Fig. 2. Expression of iron regulatory proteins in synoviums of RA and HC. a. Representative immunohistochemical staining. b. Quantitative evaluation of FTH1, FTL, TFRC and FPN1 expression between the two group (N = 10 RA; N = 10 HC; two different area from each sample were collected for statistics). *P < 0.05; **P < 0.01; ***P < 0.001; by Student's t test (mean ± SD). HC, healthy control. RA, rheumatoid arthritis.

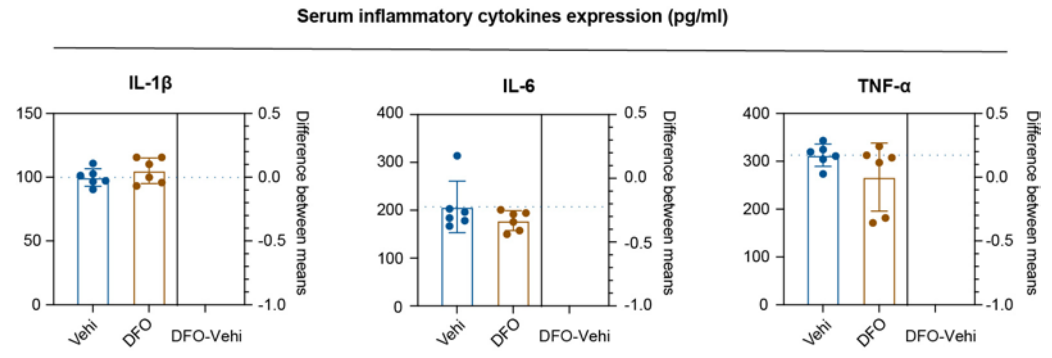

Supplementary Fig. 3. Serum levels of IL-1β, IL-6, and TNF-α were detected by ELISA assay in STIA model with DFO administration. N = 6. Data were Mean ± SD and analyzed by Student's t test. ns, not significant. STIA, K/BxN serum-transfer induced arthritis. DFO, Desferrioxamine.

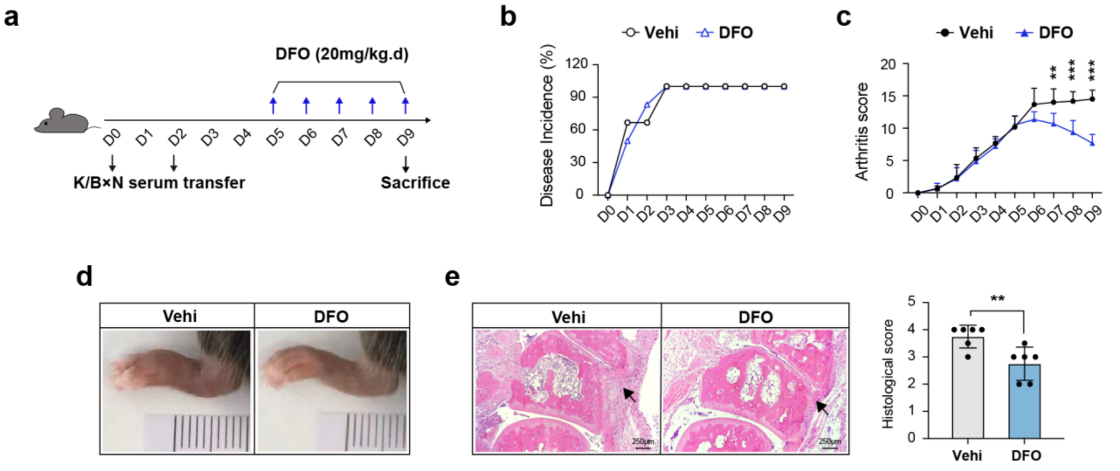

Supplementary Fig. 4. (a) The timeline design of STIA models with DFO therapeutic administration. (b-c) Disease incidence and arthritis severity scores were determined daily after modeling and statistically analyzed for 9 days. N = 6 in each group, $^{**}P < 0.01$; $^{***}P < 0.001$; by Student's t test (mean ± SD). (d) Gross morphology of the arthritis. (e) STIA mice with DFO treatment daily were sacrificed at 9 d. Histological sections of paws were collected for H&E evaluation (the pathologic change emphasized by black arrowhead). N = 6 in each group. $^{**}P < 0.01$ vs Vehi group by Student's t test (mean ± SD). STIA, K/BxN serum-transfer induced arthritis. DFO, Desferrioxamine.

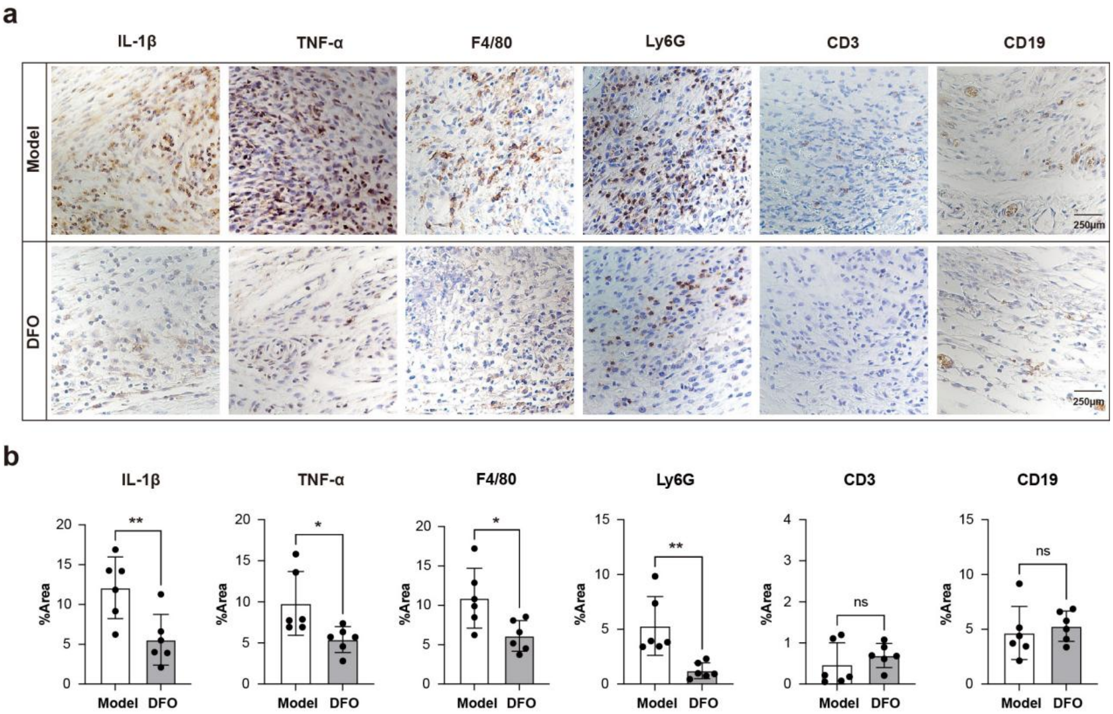

Supplementary Fig. 5. (a) Expression of the key inflammatory cytokines and infiltrated immune cells in synoviums of STIA after DFO therapeutic treatment was detected by immunohistochemical staining. (b) Immunohistochemical quantification. **P* < 0.05; ***P* < 0.01; ns, not significant, by Student's t test (mean ± SD). STIA, K/BxN serum-transfer induced arthritis. DFO, Desferrioxamine.

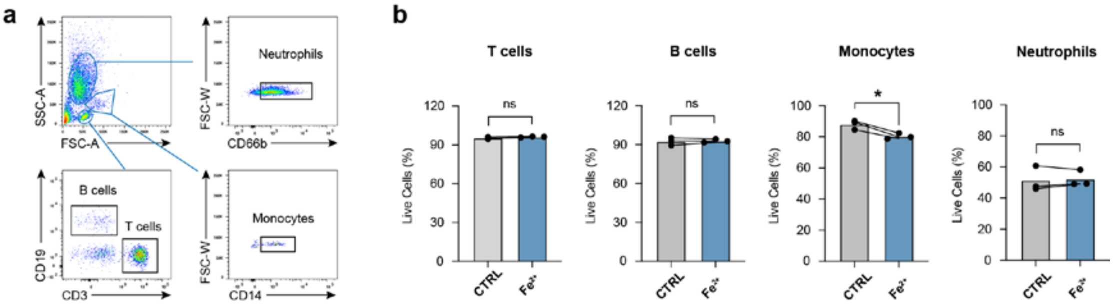

Supplementary Fig. 6. Heterogeneous viability of peripheral blood mononuclear cells (PBMCs) in response to high-iron stimulation. (a) Gating strategy for T cells ($CD3^+CD19^-$), B cells ($CD3^-CD19^+$), monocytes ($CD14^+$), and neutrophils ($CD66b^+$). (b) General survival and death of PBMCs celltypes with 500μM $FeSO_4$ stimuli for 24h was analyzed by Fixable Dye eFluorTM 780. N = 3. **P* < 0.05 compared with the CTRL group by paired t-test (mean ± SD). ns, not significant.

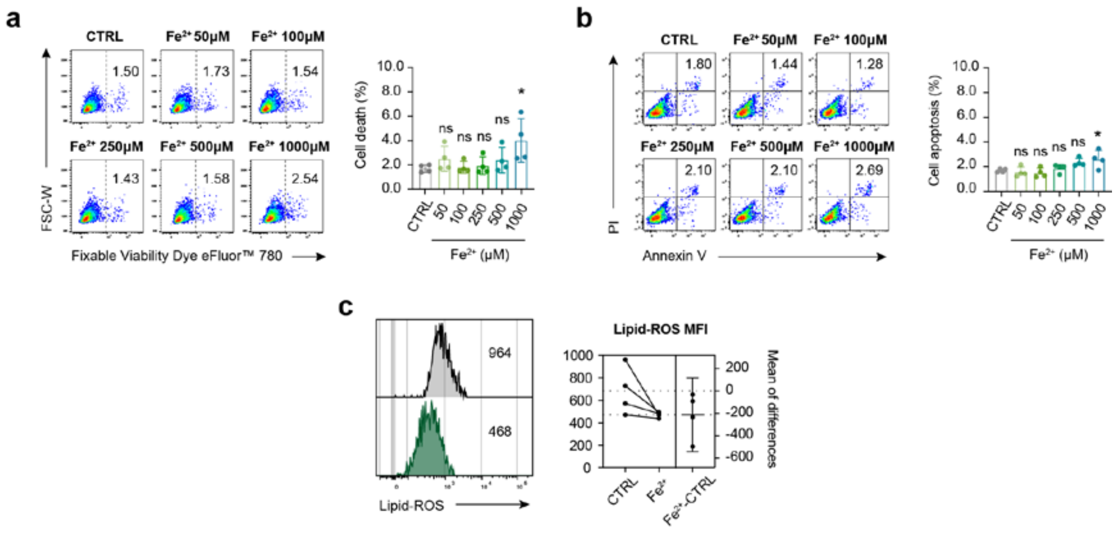

Supplementary Fig. 7. Effects of ferrous iron on general cell death, apoptosis and lipid peroxidation of RA-FLSs. (a) Dose-dependent general cell death of FLSs stimulated with $FeSO_4$ for 24h was analyzed by Fixable Dye eFluorTM 780. N = 4. **P* < 0.05 compared with the CTRL group by one-way ANOVA. (b) FLSs apoptosis in response to dose-dependent $FeSO_4$ for 24h was analyzed by Annexin V/PI. N = 4. **P* < 0.05 compared with the CTRL group by one-way ANOVA. (c) Lipid peroxidation changes with BODIPY™ 581/591 C11 staining in 500μM $FeSO_4$ treated FLSs was measured by flow cytometry. N = 4. not significant with the CTRL group by paired t-test.

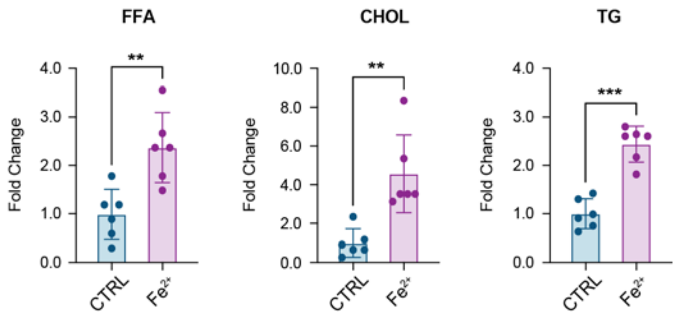

Supplementary Fig. 8. Total FFA, CHOL, and TG in RA-FLSs following 24h coculture with 500μM $FeSO_4$ were detected by relative quantification kits (N=6), ***P* < 0.01; ****P* < 0.001 compared with CTRL group by Student's t test (mean ± SD).

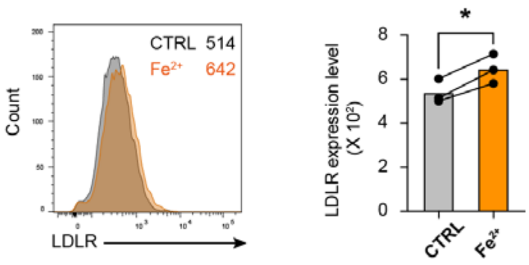

Supplementary Fig. 9. LDLR expression change in FLSs in response to iron overloading. FLSs stimulated with 500μM $FeSO_4$ for 24h was analyzed by by flow cytometry. N = 3. *$P < 0.05$ compared with the CTRL group by paired t-test.

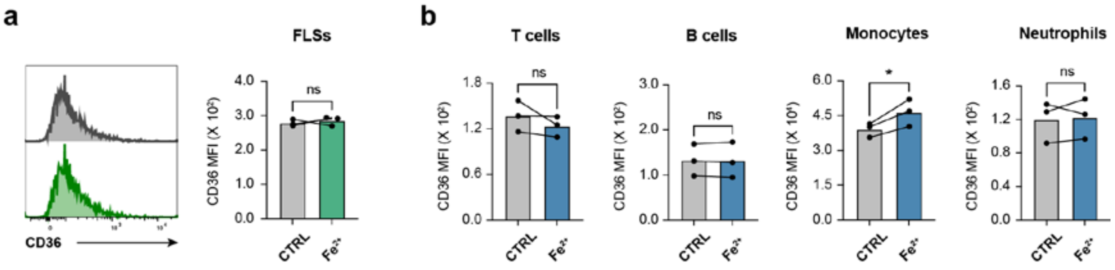

Supplementary Fig. 10. The expression of CD36 on different cell subtype under iron-overloading microenvironment. CD36 expression on FLSs (a) and PBMCs (b) stimulated with 500μM $FeSO_4$ for 24h was analyzed by flow cytometry. N = 3. *$P <$ 0.05 compared with the CTRL group by paired t-test.

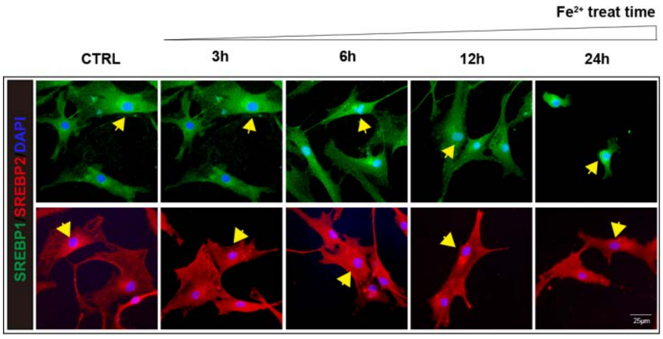

Supplementary Fig. 11. The time-course nuclear translocation of SREBP1 (Green) and SREBP2 (Red) upon $FeSO_4$ stimulation (500μM) for indicated time period was evaluated with immunofluorescence, the blue (DAPI) indicated the nucleus. N=3.

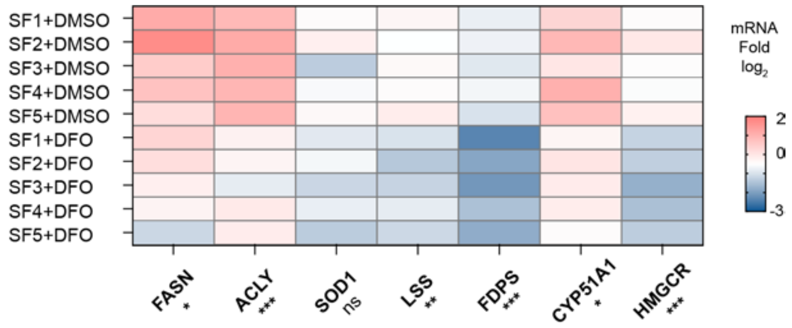

Supplementary Fig. 12. qPCR array revealed the expression of lipid synthetase in RA-FLSs by addition of DFO to block excess iron in synovial fluid. N = 5 for each group. **P* < 0.05; ***P* < 0.01; ****P* < 0.001; ns, not significant in Student's t test (mean ± SD). SF, synovial fluid; DFO, Desferrioxamine.

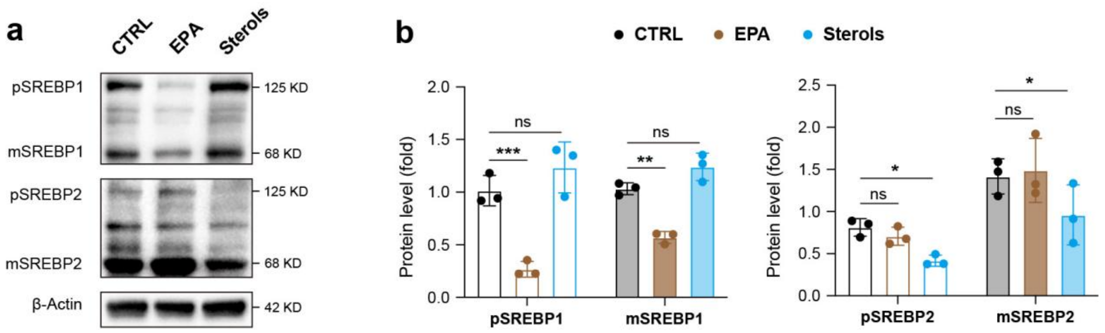

Supplementary Fig. 13. EPA specifically mediated the activation and cleavage of SREBP-1, while SREBP2 was specifically regulated by sterols. (a) Western blots analysis of SREBP1 and SREBP2 activation and cleavage in FLSs incubated with EPA (10μM, Selleck, S6476) and sterols (1μg/ml 25-hydroxycholesterol plus 10μg/ml cholesterol, Selleck, S8091 and Selleck, S4154) for 24h. (b) Quantitative statistical analysis of SREBP1 and SREBP2 activation. N = 3. **P* < 0.05, ***P* < 0.01, ****P* < 0.001 versus CTRL group by two-way ANOVA (mean ± SD), ns: not significant. EPA, eicosapentaenoic acid.

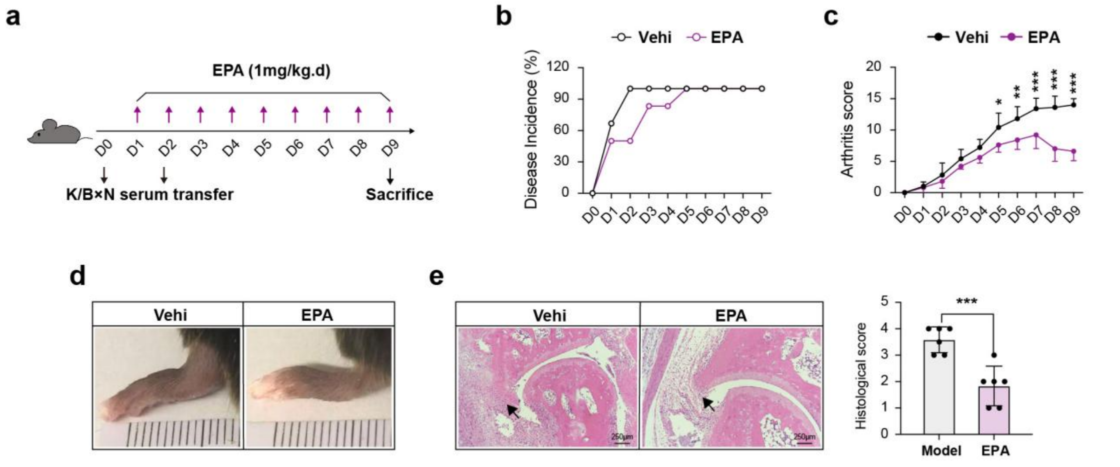

Supplementary Fig. 14. EPA can effectively inhibit the progression of arthritis in STIA mice. (a) The timeline design of STIA models with EPA administration. (b-c) Disease incidence and arthritis severity scores were determined daily after modeling and statistically analyzed for 9 days. N = 5 in each group, **P* < 0.05; ***P* < 0.01; ****P* < 0.001; by Student's t test (mean ± SD). (d) Gross morphology of the arthritis. (e) STIA mice with EPA injection daily were sacrificed at 9 d. Histological sections of whole paws were collected for H&E evaluation. (the pathologic change emphasized by black arrowhead). N = 5 in each group. ****P* < 0.001 vs Vehi group by Student's t test (mean ± SD). STIA, K/BxN serum-transfer induced arthritis. EPA, eicosapentaenoic acid.

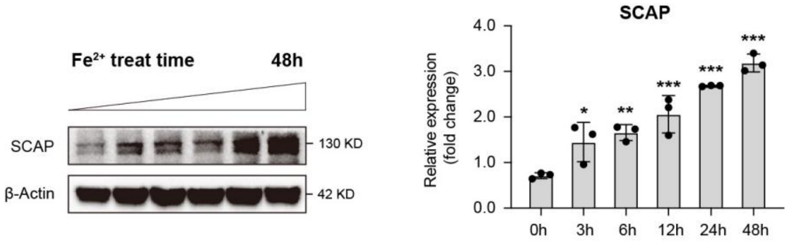

Supplementary Fig. 15. Western blot data illustrated the time-dependent upregulation of SCAP protein in iron-treated FLSs. N = 3. **P* < 0.05; ***P* < 0.01; ****P* < 0.001 versus NC group in Student's t test (mean ± SD).

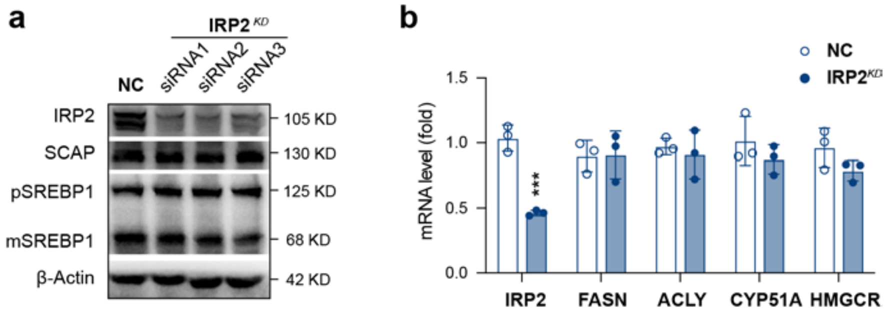

Supplementary Fig. 16. Western blot showed the effectc of IRP2 knockdown with siRNA on SCAP expression and subsequent SREBP1 cleavage-activation in FLSs. N = 3. ***P < 0.001 versus NC group in Student's t test (mean ± SD).

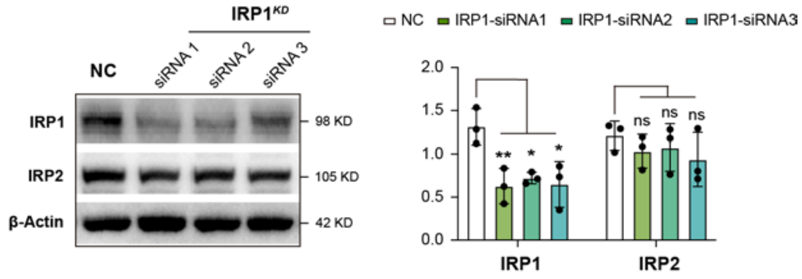

Supplementary Fig. 17. Western blot data revealed no obvious effects of IRP1 interfere on IRP2. N = 3. *P < 0.05; **P < 0.01 versus NC group in Student's t test (mean ± SD); ns, not significant.
